# Impact of Apolipoprotein L1-Associated Pregnancy Complications on Kidney Disease Phenotypes in a HIV-Associated Nephropathy Mouse Model

**DOI:** 10.34067/KID.0000001253

**Published:** 2026-07-02

**Authors:** Zhenzhen Wu, Timur Azhibekov, Chunfa Huang, Jane K. Nguyen, Korey R. Bartolomeo, Oliver Wessely, John F. O'Toole, John R. Sedor, Leslie A. Bruggeman

**Affiliations:** 1 Department of Heart, Blood and Kidney Research, Cleveland Clinic Research, Cleveland, Ohio; 2 Department of Pediatrics, Cleveland Clinic Children's, Cleveland, Ohio; 3 Case Western Reserve University School of Medicine, Cleveland, Ohio; 4 Department of Pathology and Laboratory Medicine, Diagnostics Institute, Cleveland Clinic, Cleveland, Ohio; 5 Department of Kidney Medicine, Cleveland Clinic, Cleveland, Ohio

**Keywords:** apolipoprotein L1 (APOL1), CKD, glomerulosclerosis, HIV nephropathy, kidney development, podocyte

## Abstract

**Key Points:**

- G0 coexpression did not improve CKD outcomes in a mouse model of HIV-associated nephropathy.
- Apolipoprotein L1 is expressed in the placenta, and risk variants are associated with preeclampsia, low birth weight, and reduced podocyte and glomerular density.
- Apolipoprotein L1 kidney disease risk may be mediated by apolipoprotein L1-associated birth complications negatively affecting kidney development.

**Background:**

Apolipoprotein L1* (APOL1)* polymorphisms are a significant risk factor of kidney disease including HIV-associated nephropathy (HIVAN), but a comprehensive understanding of the pathogenic mechanism remains unclear.

**Methods:**

*APOL1* transgenic models expressing *APOL1*-G0 and the kidney disease risk–associated *APOL1*-G1 variant were intercrossed with the Tg26 mouse model of HIVAN. Because all these transgenic models are hemizygous, crosses produced offspring of four genotypes: wild-type (WT) mice, *APOL1*, Tg26, and dual transgenic mice expressing both *APOL1* and Tg26 transgenes. Kidney function, pathology, and transgene expression patterns were examined in addition to podocyte and glomerular densities.

**Results:**

The WT and *APOL1* G0 or G1 mice did not develop kidney disease, whereas Tg26 and dual transgenic mice developed severe kidney disease consistent with the Tg26 HIVAN phenotype. The outcome of the G1/Tg26 intercross was expressed during pregnancy with neonatal complications that included a preeclampsia-like phenotype, low fetal/placental weight ratios, low birth weights, and fewer dual transgenic offspring that survived to adulthood. Some adult offspring from the G1/Tg26 intercross also had reduced podocyte densities and fewer glomeruli. This included the WT and *APOL1*-G1 mice, suggesting a systemic effect of the preeclampsia phenotype on the entire litter. The G0/Tg26 intercross also demonstrated kidney pathology and albuminuria similar to the G1/Tg26 intercross.

**Conclusions:**

Coexpression of G0 in the Tg26 mouse model was not protective against kidney disease. In addition, the G1-associated pregnancy complications, reduced podocyte and glomerular densities, suggest a second mechanism by which *APOL1* risk variant expression may affect long-term kidney function.

## Visual Abstract

**Figure:**
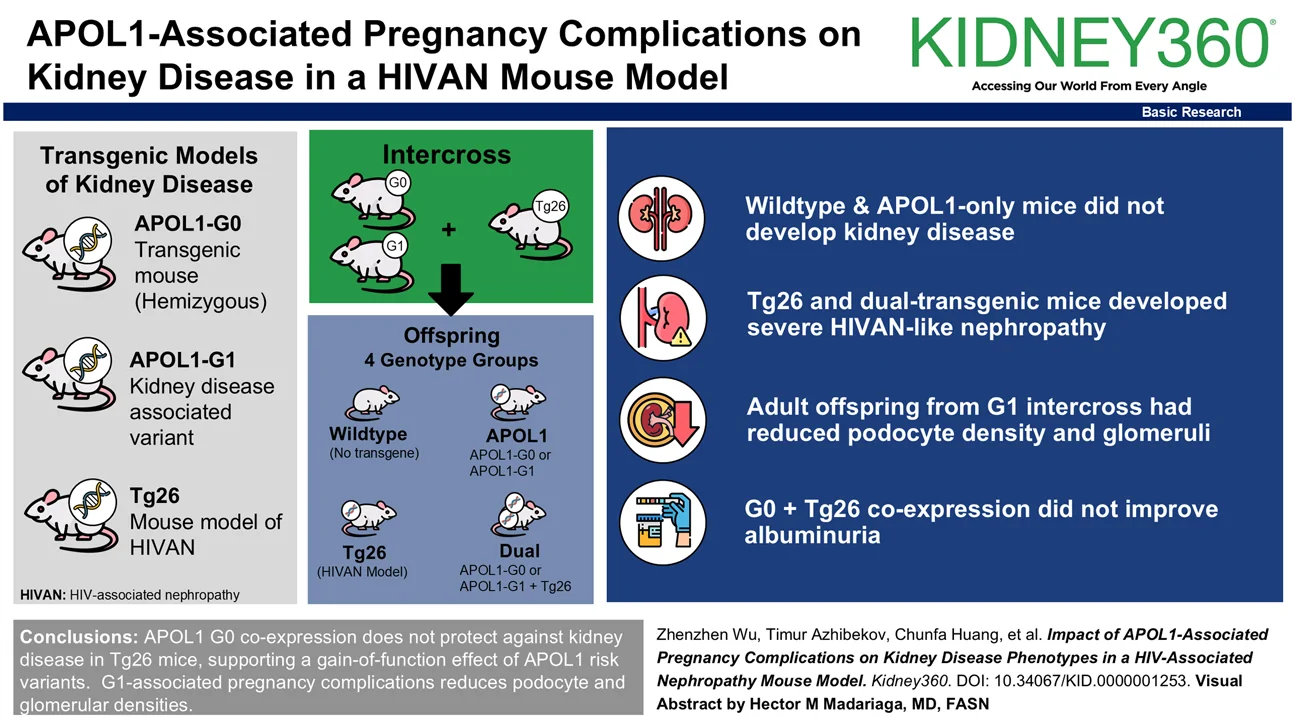

## Introduction

Allelic variants in apolipoprotein L1 (APOL1, gene name *APOL1*) contribute a significant risk of CKD,^1–3^ but a comprehensive understanding of the mechanism for kidney injury is lacking. The current theory is that the *APOL1* risk alleles, known as G1 and G2, when inherited as a recessive trait result in a susceptibility to CKD that require an additional (second-hit) stressor to induce disease.^4^ APOL1 kidney disease has been associated with a spectrum of kidney disease phenotypes that includes FSGS, a process initiated by injury or loss of podocytes. Several potential mechanisms of podocyte loss have been attributed to expression of *APOL1* risk variants; however, a consensus on the cellular pathway intersected by APOL1 that leads to cell injury or cell death is not clear. Although numerous studies report podocyte cytotoxicity with *APOL1* risk variant expression, it remains unresolved why risk is inherited as a recessive trait, an inheritance pattern more typically mediated by loss-of-function mutations.^5,6^ Another outstanding issue in the pathogenic mechanism regards the variability in APOL1 kidney disease presentation, which has led to speculation on numerous potential confounders contributing to kidney disease severity and rate of progression.^7^

Kidney disease is not the only condition associated with *APOL1* risk variants. Compelling evidence now associates the same *APOL1* risk genotypes with pregnancy complications including preeclampsia and low birth weight.^8–10^ This risk is associated with the *APOL1* genotype of the child, not the mother.^11–14^ Although preeclampsia frequently results in preterm delivery and low birth weights, even term children with *APOL1* high risk genotypes from preeclamptic pregnancies were small for gestational age.^8^ This would indicate that fetal *APOL1* high-risk genotypes fundamentally may cause an adverse intrauterine environment leading to placental insufficiency, an underlying cause for preeclampsia, prematurity, and low birth weight. Independent of *APOL1* genotype, both prematurity or low birth weight are considered risk factors for CKD later in life from low nephron endowment.^15^ Although evidence now indicates that individuals with *APOL1* high-risk genotypes are more likely to have experienced birth complications than individuals with *APOL1* low-risk genotypes, it remains unclear how APOL1-associated birth complications may contribute to APOL1’s role in future CKD risk.^16^

The goal of this study was to compare the functional impact of the nondisease *APOL1*-G0 allele versus the *APOL1* risk variant G1 on the presentation of HIV-associated nephropathy (HIVAN). The CKD with the strongest association with *APOL1* genotype is HIVAN, one of the few APOL1 kidney diseases in which the second hit stressor for CKD is known (HIV infection).^17^ Using intercrosses between the Tg26 mouse model of HIVAN with transgenic mice expressing either *APOL1*-G0 or G1, we sought to evaluate *APOL1* variant-dependent differences in kidney function, pathology, and *APOL1* expression. Possible results could either support a protective role for G0 in mitigating the HIVAN phenotype or alternatively a role of *APOL1* risk variants worsening the HIVAN phenotype. However, the outcome of this study was confounded by an overriding event, preeclampsia, which occurred in the G1 risk variant intercrosses. Although this confounding event complicated interpretation of outcomes on HIVAN, it provided additional insight into the role of APOL1-associated pregnancy complications on nephron endowment and podocyte density, which may contribute to an increased CKD risk attributable to *APOL1* genotype.

## Methods

### Mouse Models

Animal studies adhered to the National Institutes of Health Guide for the Care and Use of Laboratory Animals and were approved and monitored by the Cleveland Clinic Institutional Animal Care and Use Committee. Humane end points were established for this study with veterinary guidance and are described in Supplemental Methods. The Tg26 mouse model, maintained on the FVB/N background, was used as a model of HIVAN and has been previously described.^18^ Mice expressing human bacterial artificial chromosomes (BACs) for *APOL1*-G0, G1, or G2 were obtained on a 129/B6 background^19,20^ and have been described including identification of the transgene insertion site for the BAC-*APOL1*-G1.^21^ We determined the transgene insertion site for the BAC-*APOL1*-G0 line (see Supplemental Methods). All BAC *APOL1* transgenic lines were backcrossed >10 generations to FVB/N before intercrossing with Tg26 to eliminate the known mouse genetic background effects on the severity and penetrance of the Tg26 phenotype.^22–24^ On the FVB/N background, the G1 lifespan was shortened (see results) which required interventional euthanasia of all G1 mice by 100 days of age; therefore, the kidney disease study end point was set at 90 days so G1 mice could complete the study. *APOL1*-G2 BAC mice were not used for intercrosses because this line has lower levels of *APOL1* expression compared with the G0 and G1 expressing lines.^25,26^ BPs during pregnancy were recorded using tail cuff plethysmography as previously described (see Supplemental Methods).^27^

### RNA *In Situ* Hybridization and Immunofluorescence

Gene expression of *APOL1*, *Nphs1*, and *CD31/Pecam* was examined by mRNA *in situ* hybridization using the manual RNAscope method (ACD Bio) as previously described.^28^ Protein expression of APOL1 and CD31/Pecam1 was detected by immunofluorescence in formalin-fixed, paraffin-embedded sections as previously described.^25^ Specificity of the APOL1 antibody was confirmed using control samples in which APOL1 was absent (*i.e*., wild-type [WT] mice). Details are in Supplemental Materials.

### Renal Function and Pathology

Renal function was examined by urinary albumin-to-creatinine ratios using commercial assays for albumin (QuantiChrom Albumin assay, BioAssay Systems) and an enzymatic creatinine assay (Creatinine Mouse Assay, Crytal Chem) with kit provided controls and recommended methods. Renal pathology was scored from periodic acid–Schiff-stained slides by a pathologist who was blinded to the identity of study groups. The number of total glomeruli and glomerular features including segmental sclerosis, global sclerosis, glomerular collapse, and solidified glomeruli was counted. The degree of immune cell infiltrates (interstitial inflammation) and degree of tubulointerstitial damage reflecting tubule dilatation and microcystic changes were scored using a semiquantitative scale (zero: none, one: <10% affected, two: 11%–25% affected, three: 26%–50% affected, four: >50% affected). Fibrosis was visualized using sirius red staining, quantified by bright field image analysis and reported as percent area stained (details in Supplemental Methods).

### Podocyte and Glomerular Density

Podocyte and glomerular densities were determined using validated stereologic methods in single sections for adult mice completing the study. A method developed by Venkatareddy *et al*.^29^ was used to estimate podocyte densities and glomerular volumes as we have used previously in mouse models.^27,30^ For podocyte counting, two color immunohistochemistry (ImmPRESS polymer kit, Vector Labs) was used according to manufacturer instructions for WT-1 to identify podocytes and synaptopodin to define glomerular tufts areas in 4-*µ*m sections of formalin-fixed paraffin-embedded kidney tissue. Bright field images were captured and processed using ImageJ to quantify glomerular areas and podocyte numbers as previously described.^30^ Nephron number was estimated in periodic acid–Schiff-stained, sagittal sections by counting the number of glomeruli per a cortical area, averaging counts from ten low power fields. This method of counting glomerular densities is only an approximation for nephron number which requires inclusion of kidney volume to be accurate. Kidney volume data were not available for this study.

### Statistics

Power calculations were based on SDs on renal function and pathology data from previous studies examining Tg26 renal disease outcomes, with an *n*=8 mice per group per sex. Other than weight, there were no significant differences between sexes, and all other results are reported as combined males and females. Comparisons of independent groups were by the Welch *t* test with *P* values adjusted for multiple comparisons using Bonferroni correction. Comparisons of more than two groups were by one-way ANOVA with a *post hoc* test for multiple comparisons using Tukey honestly significant difference. Histopathology scoring data were not normally distributed and were analyzed using the Kruskal–Wallis test with *post hoc* Dunn test and Bonferroni correction. Survival analyses used the Kaplan–Meier method with the log-rank test. Animals that were subject to interventional euthanasia for reasons other than a kidney disease end point were excluded from the study and not included in survival curves. Differences in genotype frequencies were evaluated with a Chi-square goodness-of-fit test. All comparisons used *P* < 0.05 for significance and sample sizes are reported in tables and figures. Analyses used Microsoft Excel or Statistical Kingdom (statskingdom.com) which uses *R* programming.

## Results

The BAC-*APOL1* mouse models express large transgenes spanning the entire *APOL1* genomic region and replicate native *APOL1* gene expression patterns. The Tg26 mouse models carries a subgenomic HIV-1 proviral DNA and expresses most HIV proteins but is not infectious and does not produce virus. The Tg26 mouse model is therefore not a model of HIV infection but models the chronically infected cell state in which expression of HIV proteins in kidney epithelial cells causes pathology.^31^ The Tg26 mouse model authentically replicates the disease course and kidney pathology seen in human HIVAN,^18^ but the kidney disease phenotype is highly dependent on the mouse genetic background.^22–24^ Therefore, all studies used mice on the susceptible FVB/N background, which required backcrossing the BAC-*APOL1*-G0 and BAC-*APOL1*-G1 mouse models to FVB/N for >10 generations to assure preservation of the HIVAN phenotype in the intercross. After completing the backcross to FVB/N, the G1 line experienced a decrease in lifespan and is described in Supplemental Methods and Supplemental Figure 1A. Although the cause of early death remains uncertain, several causes have been ruled out including kidney disease (Supplemental Figure 1B) and hydrocephalus, a phenotype others have reported^21^ on the C57BL/6 background (Supplemental Figure 1, C–G). In addition, both the Tg26 and the BAC-*APOL1* lines are maintained as hemizygotes (transgene carried on only one chromosome) because homozygotes are not viable or sustainable in breeding. Because mice do not have an ortholog for human *APOL1* (*i.e*., are *APOL1* null) and *APOL1* gene expression is only from the transgene, mice expressing a single allele of the G0 or G1 transgene are similar to humans who are homozygous for G0 or G1. Therefore, the G1 transgenic mice replicate the human equivalent of an *APOL1* high-risk genotype.

### APOL1-G1 and HIVAN Cross Affected Survival and Birthweight

To examine the effects of G0 and the risk variant G1 on the pathology and disease course of HIVAN, we intercrossed the BAC-*APOL1*-G0 and G1 with the Tg26 mouse model of HIVAN. Because these lines are hemizygous, the intercross is predicted to produce offspring with four possible genotypes in equal ratios: 25% WT, 25% APOL1 (either G0 or G1), 25% Tg26, and 25% dual transgenic (Figure 1A). The G0/Tg26 crosses (*n*=10 litters) produced the expected ratios of offspring genotypes, whereas the G1/Tg26 crosses (*n*=11 litters) produced fewer Tg26 and dual transgenic pups (Figure 1B). In addition to fewer Tg26 and dual transgenic offspring, a greater number of dual transgenic pups were stillborn or died before weaning (day 21; Figure 1, C and D). The pups that died exhibited a similar failure-to-thrive phenotype previously reported in the Tg26 line for pups that are homozygous for the HIV transgene and represents a severe presentation of the Tg26 phenotype.^32,33^ This observation may suggest that copresence of *APOL1*-G1 with the Tg26 transgene exacerbated the Tg26 phenotype, which was not seen in the G0/Tg26 cross. This is consistent with a previous study similarly intercrossing BAC-*APOL1* mice with Tg26 but on the 129SvJ background, which observed that adult G1 dual mice had more severe kidney disease.^26^

**Figure. 1:**
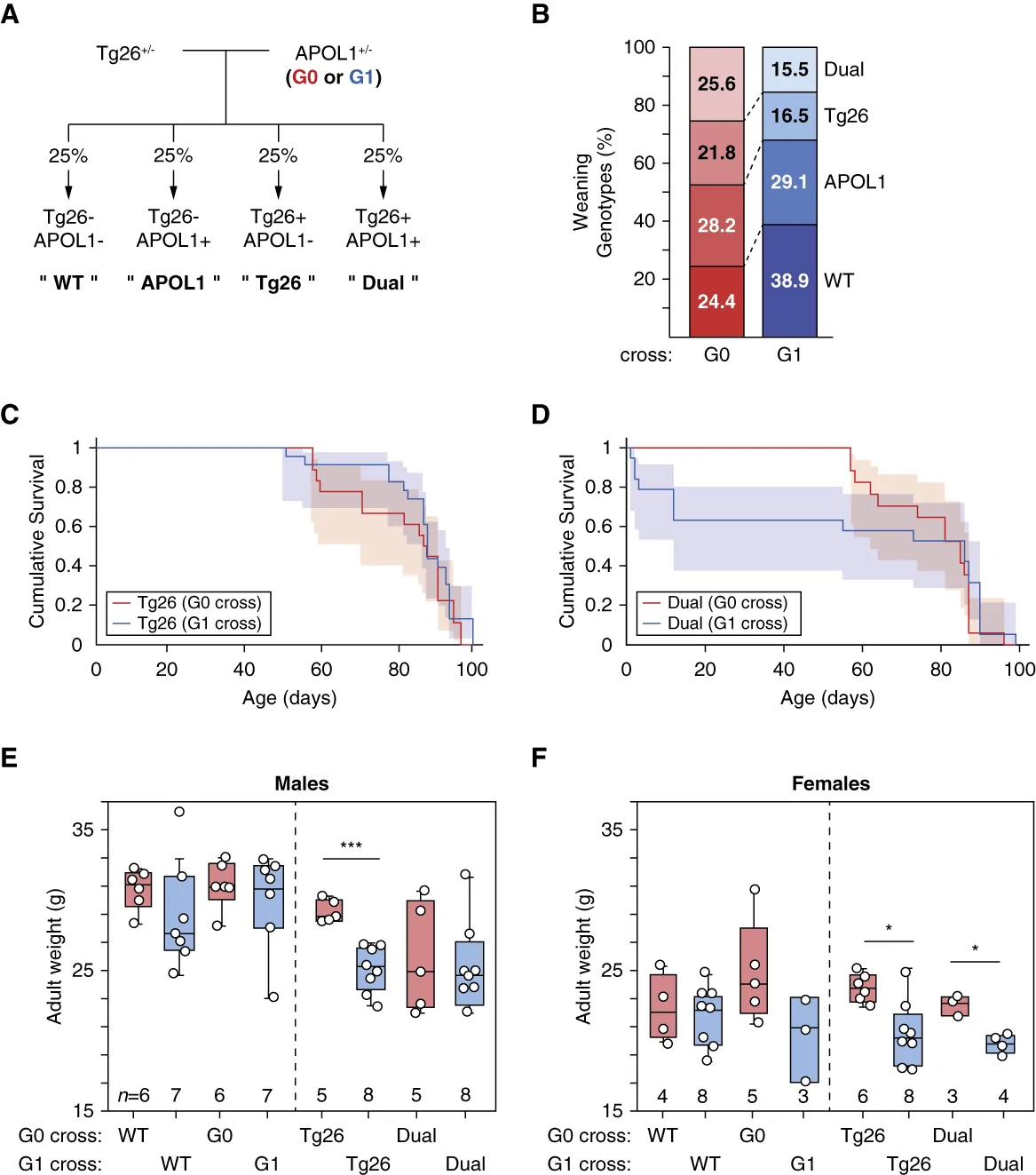
***APOL1*-G1 transgenic mice intercrossed with a Tg26 HIVAN mouse model resulted in loss of fetuses/pups and underweight adults.** (A). Scheme for crossbreeding the Tg26 mouse model of HIVAN with the BAC-*APOL1*-G0 and -G1 lines showing the expected genotype frequencies in the matings. The Tg26 and both G0 and G1 transgenic lines are maintained as hemizygotes (*i.e*., transgene is carried on only one chromosome) thus offspring genotypes will include non-transgenic WT mice, mice expressing only the Tg26 transgene, mice expressing only the *APOL1*-G0 or G1 transgene, or mice expressing both Tg26 and *APOL1* transgenes referred to as dual transgenics. (B) At weaning, the prevalence of offspring genotypes for the G0/Tg26 cross produced expected ratios (chi-squared test [3, *n*=78] =0.67, *P* = 0.89). The prevalence of offspring genotypes for the G1/Tg26 cross did not produce expected ratios (chi-squared test [3, *n*=103] =14.51, *P* = 0.002), with reduced numbers in Tg26 and dual transgenic mice. (C) Kaplan–Meier curves comparing survival of Tg26 mice from the G0/Tg26 and G1/Tg26 crosses, which were not significantly different (log-rank test *P* = 0.4). Curves reflect mice reaching a kidney disease end point or the study end point at 90 days of age. (D). Kaplan–Meier curves comparing survival of dual transgenic mice from the G0/Tg26 and G1/Tg26 crosses. The dual transgenic mice expressing G1 more frequently experienced neonatal deaths compared with G0 (RR=2.42, 95% CI, 1.57 to 3.73, *P* = 0.0001), although survival curves were not significantly different (log-rank test *P* = 0.6). (E and F) Weights of adult mice at study end point for both males (E) and females (F). Weights were not available on all mice in the study, sample sizes (*n*) for each are shown on graph. Box plots show means with interquartile range and 95% CIs: *P < 0.05, ***P < 0.001 by *t* test with Bonferroni correction. APOL1, Apolipoprotein L1; BAC, bacterial artificial chromosome; CI, confidence interval; HIVAN, HIV-associated nephropathy; RR, relative risk; WT, wild-type.

In addition to the *in utero* and postnatal death of pups, fetal weights during the last week of gestation at embryonic day (e) 15.5 and e18.5 were significantly lower in G1 litters compared with G0 litters (Supplemental Figure 2A). Near term (e18.5) fetal to placental weight ratios (an indication of placental insufficiency) in G1 litters were half of that observed for G0 litters (Supplemental Figure 2B). This resulted in average birth weights of pups from the G1 being significantly lower than G0 pups (Supplemental Figure 2C). This difference was not driven by litter size because there was no significant difference in litter size between G0/Tg26 and G1/Tg26 intercrosses (Supplemental Figure 2D). This impact on weight persisted into adulthood in some genotypes, with significant differences in weights in Tg26 males and Tg26 and dual transgenic females when comparing the G0/Tg26 and G1/Tg26 crosses (Figure 1, E and F).

### *APOL1* Expression in Placenta and Preeclampsia

Pregnancy complications associated with fetal *APOL1* risk variants have been reported in humans including preeclampsia, low birth weight, and prematurity.^8–11,13,14^ In the G1 mice on the FVB/N background, all adult female mice examined did not have hypertension before pregnancy, but had significantly elevated BPs during pregnancy, which resolved postpartum (Figure 2A). The severity of the hypertension varied, and approximately 25% of dams progressed to eclampsia (and were euthanized), with the remaining dams surviving to deliver and wean their litters. G0 mice did not have elevated BP during pregnancy or present with eclampsia. This preeclampsia phenotype, including hallmarks of preeclampsia such as elevated soluble fms-like tyrosine kinase-1/placental growth factor ratios, has been previously reported in this BAC-*APOL1*-G1 line on the 129SvJ background.^34^ This study also reported G0 mice do not have preeclampsia and is consistent with our observations.

**Figure 2:**
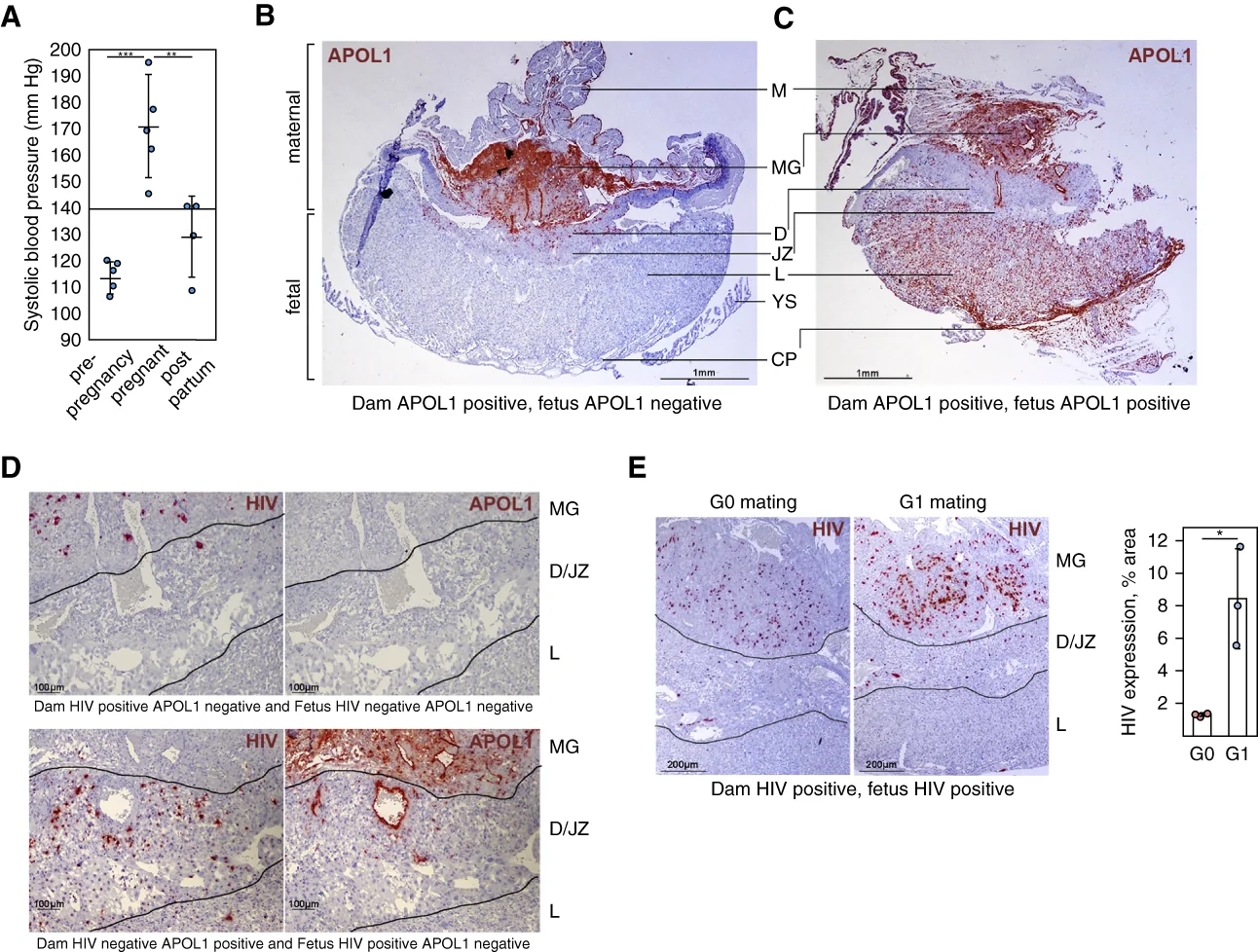
**BAC *APOL1* mice expressed *APOL1* and HIV transgenes in both maternal- and fetal-derived portions of the placenta and G1 mice developed a preeclampsia-like phenotype.** (A) Maximum systolic BP obtained in each recording period in *APOL1*-G1 expressing mice. BP were recorded every other day for 1 week before pregnancy (pre-pregnancy), during pregnancy, and 1 week postpartum. Data show means±SD; by ANOVA, adjusted for multiple comparisons (Tukey HSD) **P = 0.004, ***P < 0.001. (B and C) Expression of *APOL1* by mRNA *in situ* hybridization in near term placentas (e18.5). To clearly visualize *APOL1* expression originating from the maternal and fetal derived-components, two placentas are shown from an *APOL1*-G0 positive dam, one placenta was from a fetus that was *APOL1* negative (B) and one placenta was from a fetus that was *APOL1* positive (C). *APOL1* expression was abundant the maternal-derived M, MG, and D. *APOL1* expression was abundant in the fetal-derived JZ, L, CP, and vasculature of the umbilicus and YS. Visualization of specific cell types is presented in higher magnification in Supplemental Figure 2. (D) RNA *in situ* hybridization for the Tg26 HIV transgene with the *APOL1* transgene in serial sections (e16.5 placentas) comparing dams and fetuses of different genotypes. HIV-positive cells were detected in both maternal and fetal compartments. In serial sections, there were no cells expressing both transgenes because the *APOL1-* and HIV-positive cells originated from different compartments of the placenta. (E) The Tg26 HIV transgene in which both the dam and fetus were HIV positive indicated the most abundant expression of the Tg26 transgene was in the maternal tissues in the MG. Quantification of HIV gene expression indicated the abundance of the HIV expressing cells in the MG was greater in the G1 cross, *P < 0.05 (*n*=3). CP, chorionic plate; D, decidua; HSD, honestly significant difference; JZ, junctional zone; L, labyrinth; M, myometrium; MG, metrial gland; YS, yolk sac.

In both BAC-*APOL1* models, *APOL1* RNA was abundantly expressed in the murine placenta in both the maternal- and fetal-derived components, which was discerned by examining combinations of dams and pups with and without *APOL1* transgenes (Figure 2, B and C). The maternal components included expression in cells of the myometrium, metrial gland and decidua, and endothelia of all vascular structures (Supplemental Figure 3A). The fetal components that express *APOL1* RNA included all trophoblast cell types, including syncytiotrophoblasts of the labyrinth; and spongiotrophoblasts, glycogen trophoblasts, and trophoblast giant cells of the junctional zone; and endothelial cells of all vascular structures include the chorionic plate and umbilicus (Supplemental Figure 3A). Similarly, in a term human placenta, *APOL1* RNA expression was abundant in all trophoblast cell types including syncytiotrophoblasts, cytotrophoblasts, and extravillous trophoblasts and all vascular endothelia (Supplemental Figure 3B). In both humans and mice, preeclampsia is initiated by failed maternal spiral artery remodeling by fetal-derived trophoblasts.^35,36^ In mice, spiral artery remodeling is mediated by the glycogen trophoblasts and trophoblast giant cells, whereas in humans spiral artery remodeling is initiated by extravillous trophoblasts.^36^ Therefore, in both the human and mouse placenta, *APOL1* RNA was expressed in critical cell types required for spiral artery remodeling.

The HIV transgene was also expressed in the placenta in both maternal and fetal compartments. The breeding strategy used *APOL1* dams and Tg26 males for all G0 matings. Owing to the pregnancy complications in the G1 crosses, some of the G1 matings used the opposite pairing of Tg26 dams and *APOL1* males. Thus, 100% of G0 pups and 86% (88 of 103) G1 pups were from dams that carried the *APOL1* transgene and not the HIV transgene. In Tg26 and dual transgenic mice, HIV was expressed in fetal-derived cells, and in the few Tg26 dams used in the study, HIV expression was also evident in maternal-derived cells (Figure 2 D). HIV expressing cells derived from either the maternal or fetal compartments were also present in the shared junctional zone but did not appear to migrate into the opposing compartment. In the few G1/Tg26 intercrosses in which the dam carried the Tg26 transgene, there appeared to be a greater number of HIV-expressing cells in the maternal compartment (metrial gland, Figure 2E). In humans, HIV-positive mothers have a higher risk of pregnancy complications including miscarriages, still births, intrauterine growth restriction, and placental insufficiency.^37,38^ Thus, this increase in HIV expression in the metrial gland may have contributed to pregnancy complications in the G1/Tg26 matings that used Tg26 dams. Most mice in the study, however, were not from Tg26 dams. The absence of *APOL1* in the dam was unlikely to have affected the pregnancy complications because preeclampsia risk is associated with the fetal, not maternal, *APOL1* genotype.^11–14^ In addition, maternal HIV expression from Tg26 dams did not appear to change study outcomes in a sensitivity analysis when offspring from Tg26 dams were excluded (Table 1), likely because of the small number of animals. Additional studies investigating this effect of G1 on increasing HIV expression in the placenta may be useful in defining a role for second-hit stressors in preeclampsia.

**Table 1:** Sensitivity analysis on effect of *APOL1*-negative Tg26 dams (*P* values)

Figure	Comparison	Genotype Group	All Dams	Tg26 Dams Removed
Figure 1 males	G0 versus G1	WT	0.18	0.35
Figure 1 males	G0 versus G1	APOL1	0.38	0.60
Figure 1 males	G0 versus G1	Tg26	0.002	0.002
Figure 1 males	G0 versus G1	Dual	0.76	0.91
Figure 1 males	Tg26 versus dual	G0	0.09	0.09
Figure 1 males	Tg26 versus dual	G1	0.24	0.69
Figure 1 females	G0 versus G1	WT	0.48	0.64
Figure 1 females	G0 versus G1	APOL1	0.11	0.11
Figure 1 females	G0 versus G1	Tg26	0.04	0.05
Figure 1 females	G0 versus G1	Dual	0.02	0.02
Figure 1 females	Tg26 versus dual	G0	0.18	0.18
Figure 1 females	Tg26 versus dual	G1	0.50	0.51
Figure 4	G0 versus G1	WT	0.02	0.02
Figure 4	G0 versus G1	APOL1	0.03	0.03
Figure 4	G0 versus G1	Tg26	0.68	0.74
Figure 4	G0 versus G1	Dual	0.12	0.16
Figure 4	Tg26 versus dual	G0	0.13	0.13
Figure 4	Tg26 versus dual	G1	0.53	0.67
Figure 5A	G0 versus G1	WT	0.13	0.21
Figure 5A	G0 versus G1	APOL1	0.002	0.01
Figure 5A	G0 versus G1	Tg26	0.10	0.40
Figure 5A	G0 versus G1	Dual	0.65	0.72
Figure 5A	Tg26 versus dual	G0	0.19	0.19
Figure 5A	Tg26 versus dual	G1	0.64	0.89
Figure 5B	G0 versus G1	WT	0.20	0.30
Figure 5B	G0 versus G1	APOL1	<0.001	0.001
Figure 5B	G0 versus G1	Tg26	0.45	0.20
Figure 5B	G0 versus G1	Dual	0.78	0.77
Figure 5B	Tg26 versus dual	G0	0.18	0.18
Figure 5B	Tg26 versus dual	G1	0.85	0.92

*t* test, *P* values adjusted by Bonferroni correction (*n*=6 comparisons). APOL1, Apolipoprotein L1; WT, Wild-type.

### *APOL1* Expression in the Kidney

In humans and mouse models, the level of *APOL1* expression is important for inducing kidney disease. In the G0, G1, and dual transgenic mice, *APOL1* was expressed in the glomerulus (podocytes, endothelia, and some parietal cells), in all vascular endothelial cells including peritubular capillaries and larger vessels, and in immune cells in infiltrates. There was a similar level and pattern of *APOL1* expression comparing G0 and G1 mice (Figure 3, A and C). In the dual transgenic mice, *APOL1* expression patterns changed reflecting the pathology of HIVAN (Figure 3, B and D). This included increased *APOL1* expression in the interstitium because of the increased presence of immune cell infiltrates. However, there was no new expression in other cell types such as tubular epithelia. Although *APOL1* expression was high in structurally normal glomeruli, *APOL1* expression decreased in structurally abnormal glomeruli in the dual transgenics from the sclerotic changes in HIVAN. Previous studies have shown glomerular injury in HIVAN includes podocyte dedifferentiation and cell death,^39,40^ which likely underlies this loss in *APOL1* expression. When quantifying total kidney *APOL1* expression, there was no significant difference between G0 and G0 dual transgenic or the G1 and G1 dual transgenics (Figure 3E). Thus, this total *APOL1* quantification reflected a composite of multiple changes including decreases in expression in injured glomeruli, increases from immune cell infiltrates, in addition to the anatomic distortion of the tubulointerstitium with cysts.

**Figure 3:**
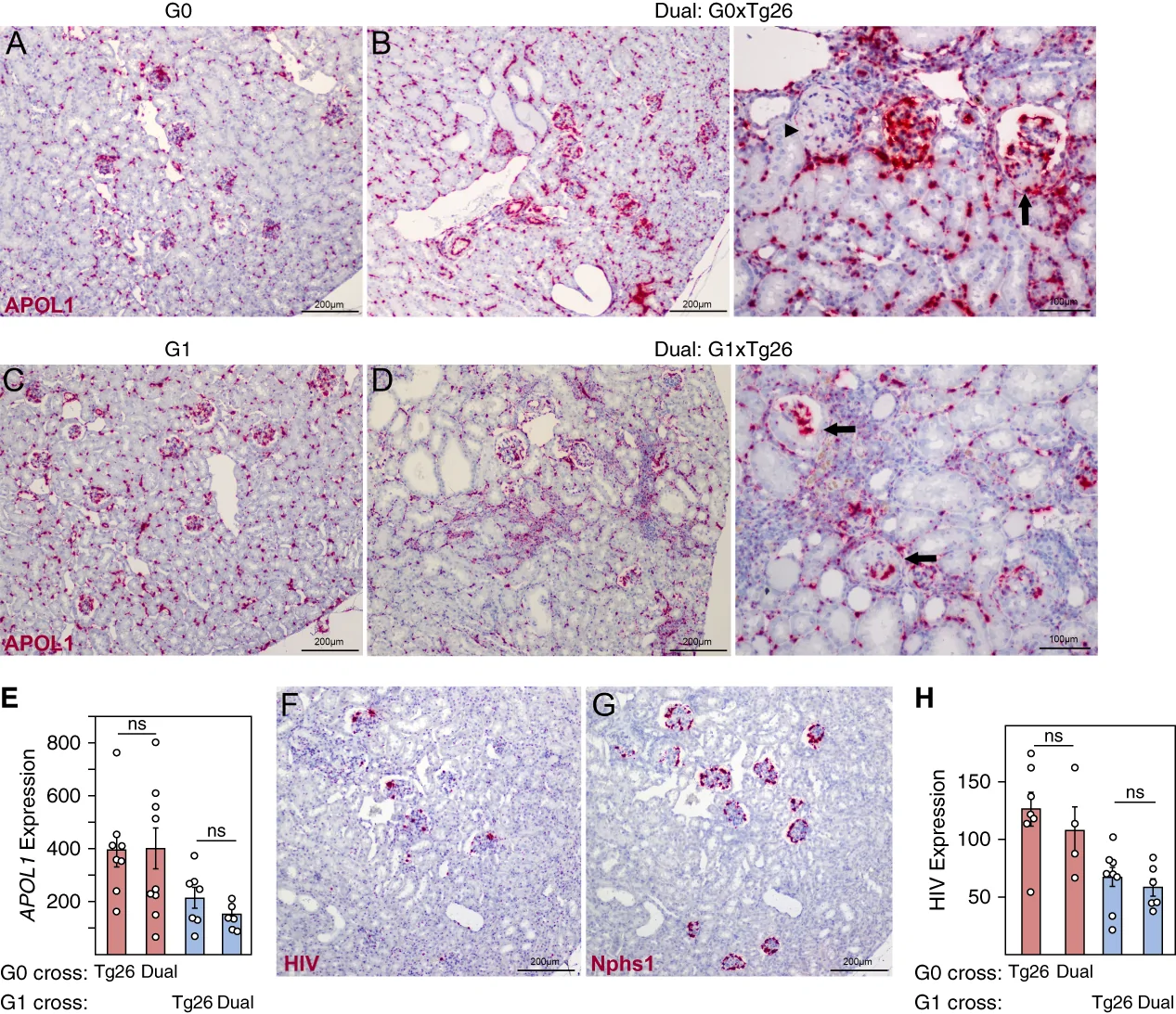
**Expression of *APOL1* and Tg26 HIV transgenes were not significantly different in the intercrosses.** Using mRNA *in situ* hybridization, expression of *APOL1*, HIV, and *Nphs1* (the gene for nephrin as a podocyte marker) were quantified by image analysis. (A). *APOL1* was expressed in G0 mice with the expected pattern in the glomerulus (podocytes, endothelial cells, and some parietal cells) and in endothelial cells of all vascular structures including veins, arteries, and peritubular capillaries. (B) *APOL1* expression in G0/Tg26 dual transgenic demonstrated reduced *APOL1* expression in diseased glomeruli (arrow indicates glomerulus with segmental sclerosis, arrow head indicates glomeruli with global sclerosis) in addition to increases in *APOL1* expression in the interstitium from immune cell infiltrates. (C and D) *APOL1* was expressed in G1 (C) and G1/Tg26 dual transgenic (D) mice in a similar pattern as G0 mice. (E) Whole-section quantification of *APOL1* expression by image analysis was not different between Tg26 or dual transgenic mice in either intercross (by *t* test). (F and G) HIV transgene expression was detected in all kidney epithelia but was most strongly expressed in podocytes (identified by staining for *Nphs1* in serial sections). Higher magnification images are shown in Supplemental Figure 4. (H) HIV gene expression quantified by whole-section image analysis did not identify significant differences between Tg26 or dual transgenic mice in either intercross (*n*=4–9 each group), although expression levels trended lower in the G1/Tg26 intercross (by *t* test).

Similar to the requirement of *APOL1* expression levels and kidney disease, previous studies have shown kidney expression of the HIV transgene in Tg26 mice also is important for disease induction.^41,42^ To determine whether HIV gene expression was altered in the crosses, expression patterns were compared between Tg26 and dual transgenic mice (Figure 3, F and G). HIV gene expression is known to occur in all epithelial cells including podocytes and tubular epithelial, with expression typically the highest in glomerular podocytes (Supplemental Figure 4). There was no significant difference in the Tg26 transgene expression between the G0/Tg26 and G1/Tg26 crosses, although expression trended lower in mice from the G1 cross (Figure 3H).

With the pregnancy complications in the G1 mice, both *APOL1* and HIV transgenes were also examined in fetal kidneys. *APOL1* expression in e18.5 kidneys was low and sporadic in cells outside glomeruli with no expression in podocytes (Supplemental Figure 5, A and B). This is consistent with a previous report that in humans, *APOL1* is not detectable during kidney development.^43^ HIV gene expression was also low and limited to podocytes, but only in the most mature glomeruli deepest in the cortex (Supplemental Figure 5, C and D). Together, these findings suggest the pregnancy phenotype in G1 mice was unlikely to have arisen from embryonic *APOL1* or Tg26 expression in the kidney.

### APOL1 Impact on Kidney Disease

The evaluation of adult CKD outcomes in the G1/Tg26 intercross was confounded by pregnancy complications that markedly decreased postnatal survival of dual transgenic offspring. This included young pups exhibiting the Tg26 failure-to-thrive phenotype, considered a severe presentation of kidney disease in the Tg26 model. These events possibly eliminated more severely affected G1 dual transgenic animals before the planned 90-day end point, potentially altered outcomes in the adult CKD phenotyping. Therefore, the adult G1 phenotyping was compromised by both low numbers of animals and a potential survivor bias. Owing to this dominant effect of the pregnancy complications, the G1 adult phenotyping has limitations from both biologic and statistical considerations. This pregnancy complication, however, was not observed in the G0/Tg26 intercross and the CKD phenotyping analysis from this intercross should be valid.

As expected in the assessment of CKD, albuminuria in all mice carrying the Tg26 transgene was significantly greater compared with WT or mice only expressing *APOL1* G0 or G1 (Figure 4). Although albuminuria in the APOL1 (G0 and G1) and WT mice from both intercrosses was within the normal range, WT mice from the G1/Tg26 intercross had significantly more albuminuria than WT mice from the G0/Tg26 intercross, and similarly, the *APOL1*-G1 mice had significantly more proteinuria than *APOL1*-G0 mice. This difference may have originated from the pregnancy complication in the G1/Tg26 intercross, which is known to impede kidney development with subsequent negative effects on kidney function in adulthood (described below). Histopathologic changes in all mice carrying the Tg26 transgene were typical for HIVAN, including glomerulosclerosis and glomerular collapse with substantial tubulointerstitial changes including tubular dilation, microcysts, casts, and immune cell infiltrates (Supplemental Figure 6). Comparisons of pathologic features between Tg26 and dual mice from the G0/Tg26 intercross had no significant differences, indicating that the coexpression of G0 had no effect on improving HIVAN in the Tg26 model (Table 2).

**Figure 4:**
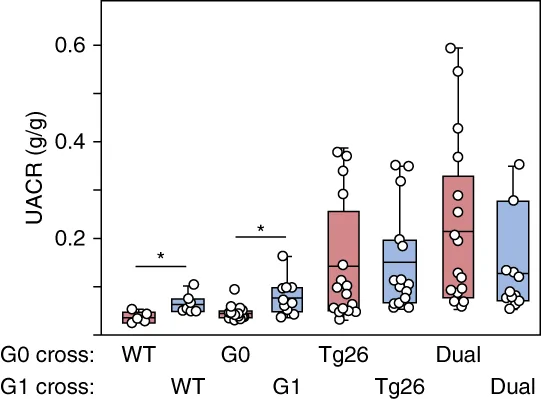
**Urinary albumin excretion was elevated in WT and *APOL1* mice from the G1/Tg26 intercross.** Albuminuria was assessed at study end point by UACR. Mice that did not carry the Tg26 transgene (WT and *APOL1* G0 or G1 mice) had normal levels of albumin excretion; however, levels were significantly higher in mice from the G1/Tg26 intercross (by *t* test, *P < 0.05). Mice that carried the Tg26 transgene (Tg26 and dual transgenics) had significantly more albuminuria, but all other genotypes were not significantly different. Urine samples were not available for all WT mice in the study (*n*=12–16 for other genotype groups). Box plots show means with interquartile range and 95% CIs. UACR, urinary albumin-to-creatinine ratio.

**Table 2:** Kidney pathology in Tg26 and dual transgenic mice from G0/Tg26 and G1/Tg26 intercross (Kruskal–Wallis test)

Genotype	Tg26 From G0 Cross (*n*=16)			Tg26 From G1 Cross (*n*=15)			Dual From G0 Cross (*n*=16)			Dual From G1 Cross (*n*=12)				
Feature	Mean	Median	Mean Rank	Mean	Median	Mean Rank	Mean	Median	Mean Rank	Mean	Median	Mean Rank	*H*	*P*
# Scored glomeruli/animal	276			246			267			275				
% Segmental sclerotic glomeruli	7.0±1.8	5.2	28.3	10.9±3.2	6.7	32.5	8.9±1.9	4.8	34	5.6±2.0	2.7	23.8	2.93	0.40
% Global sclerotic glomeruli	3.0±0.9	1.3	27.9	9.9±4.0	1.7	30.7	20.4±7.3	4.4_a_	40.1	1.6±0.8	0.3_a_	18.4	11.25	0.01
% Solidified glomeruli	1.6±0.8	0.2	35.9	0.2±0.1	0.0	27.3	0.5±0.3	0.0	28.1	0.7±0.5	0.0	28.1	3.76	0.29
% Collapsed glomeruli	0.9±0.3	0.7	34.3	0.5±0.4	0.0	23.1	1.1±0.3	0.7	37.4	0.7±0.5	0.0	23	9.45	0.02^a^
% All sclerotic glomeruli	11.3±2.7	7.5	27.9	20.9±6.8	10.0	30.9	29.8±8.6	9.0	37.3	7.9±2.9	3.0	21.9	5.83	0.12
Tubular microcysts	1.3±0.4	1.0	24.9	1.7±0.3	1.0	31.1	2.4±0.4	2.0	38.8	1.1±0.3	1.0	23.6	7.93	0.05
Interstitial inflammation	1.3±0.3	1.0	26.9	1.5±0.4	2.0	28.2	2.4±0.4	2.0	38.6	1.3±0.4	1.0	25	6.04	0.11
Fibrosis	3.4±0.9	2.4	23.4	4.7±1.3	1.8	23.9	5.1±0.8	5.13	30.1	1.8±0.6	0.8	15.1	6.01	0.11

Kruskal–Wallis test, *post hoc* Dunn test, Bonferroni correction (df=3; *n*=59 for all except fibrosis scoring *n*=47).

Medians in a row sharing subscripts are significantly different from one another.

a Pairwise comparisons did not identify statistically significant differences.

### APOL1 Impact on Podocyte and Glomeruli Density

To determine whether the pregnancy phenotype affected kidney development, podocyte density and glomeruli density (as an estimate of nephron number) were compared between all offspring from the G0/Tg26 and G1/Tg26 intercrosses. As expected, podocyte densities were lower in all mice that developed kidney disease compared with mice without kidney disease (Figure 5A). This was consistent for both the G0/Tg26 and G1/Tg26 crosses, and podocyte densities were not significantly different in Tg26 and dual mice from both crosses. However, *APOL1* mice from the G1/Tg26 cross had significantly lower podocyte densities in comparison with *APOL1* mice from the G0/Tg26 cross. Interestingly, the podocyte density in the G0 mice was higher than WT mice, an observation that was also reported for a different *APOL1*-G0 mouse model.^30^ Analysis of glomerular volumes, which are typically increased with decreasing podocyte density, exhibited the expected pattern (Figure 5B). Glomerular volumes were higher in all mice that developed kidney disease. The *APOL1* mice from the G0/Tg26 cross had the lowest glomerular volumes and were significantly lower than *APOL1* mice from the G1/Tg26 cross. Finally, as an estimate of nephron number, glomerular density (number of glomeruli per area of cortex) was determined for the WT and *APOL1* (G0 and G1) mice. Glomerular density in WT mice from the G0/Tg26 cross were significantly greater in comparison with the WT mice from the G1/Tg26 cross (Figure 5C). Because mice from the G1/Tg26 cross had lower adult weights, part of these differences may originate from smaller kidneys. Whether it was an effect of a kidney developmental issue or lower body weights, some mice from the G1/Tg26 intercross appeared to have reductions in podocyte and glomerular densities. As this included WT mice, these quantifications indicated a systemic consequence of the pregnancy phenotype affecting kidney development.

**Figure 5:**
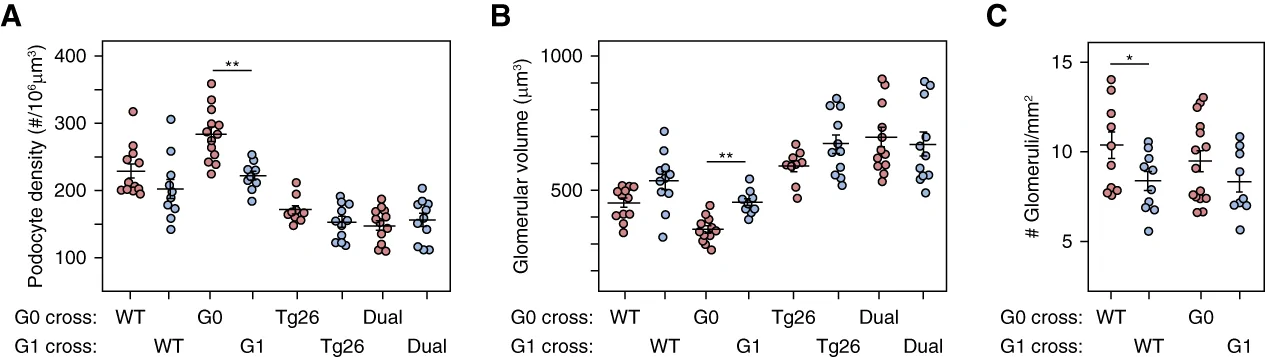
**Podocyte densities were reduced in *APOL1* mice from the G1/Tg26 intercross**. Estimates for podocyte and glomerular densities were examined in all genotypes using single-section stereologic methods. (A) Consistent with the severe FSGS of HIVAN, podocyte densities were lower in both Tg26 and dual transgenic compared with WT or *APOL1* G0 and G1 mice in both G0/Tg26 and G1/Tg26 intercrosses, with significant differences comparing *APOL1* G0 and *APOL1* G1 mice. (B) Glomerular volumes were higher in all Tg26 and dual transgenic mice compared with WT and *APOL1* G0 or G1 mice, with significant differences between *APOL1* G0 and *APOL1* G1 mice. (C) Nephron number was approximated by counting the number of glomeruli within a defined region of the cortex (*i.e*., glomerular density). WT mice from the G1/Tg26 cross had lower glomerular density compared with WT mice from the G0/Tg26 cross. Statistical comparisons were performed for the four intergenic counterpart groups and for the intragenic comparison of Tg26 and dual mice (*n*=12–16 in each group). All data are mean±SEM, *P < 0.05; **P < 0.01, by *t* test and adjusted for multiple comparisons (Bonferroni).

## Discussion

Independent of *APOL1* genotype, prematurity and low birth weight are associated with reduced kidney function and hypertension and are considered a risk factor for CKD later in life.^44,45^ Because the majority of nephrogenesis and nephron maturation occurs late in gestation, prematurity and low birth weight is associated with reductions in nephron number and low podocyte densities, resulting in a deficit in the functional reserve of the kidney.^15,46,47^ This was also observed in this study, where the importance of the pregnancy phenotype on kidney development was evident in both the WT and *APOL1* mice, which exhibited lower podocyte numbers and glomerular densities. These outcomes suggest a systemic event in the mother affecting the entire intrauterine environment, which had a negative impact on kidney development and contributed to kidney functional declines. With the growing evidence in human cohort studies associating *APOL1* risk alleles with preeclampsia, prematurity, and low birth weight,^8–10^ these APOL1-associated pregnancy complications may be contributing to the overall risk of APOL1 kidney disease later in life.

Since the original descriptions of the *APO-L* gene locus, *APOL1* is known to be abundantly expressed in the human placenta.^48,49^ Expression of *APOL1* reported here, in both humans and mice, was localized in the key cell types responsible for spiral artery remodeling. Future studies are needed to examine the function of APOL1 in trophoblasts as understanding these differences in placental pathology may help define the mechanism of placental insufficiency mediated by the *APOL1* risk variants. Because there was little to no expression of the *APOL1* or Tg26 transgenes in the developing kidney, the reductions in glomeruli and podocyte density are likely an effect of the pregnancy phenotype. Importantly, preeclampsia has never been reported for the Tg26 model and a prior study examining birth weights did not find Tg26 pups were underweight compared with WT mice.^33^ Therefore, the cause of the preeclampsia and lower birth weights in the G1/Tg26 intercross appear to be dependent on expression of the G1 transgene not the Tg26 transgene.

Owing to the pregnancy complications in the G1/Tg26 intercross, we cannot make conclusions on the impact of G1 on the adult CKD outcomes, although the failure-to-thrive phenotype in neonates would suggests G1 exacerbated the HIVAN phenotype. A previous study by Yoshida *et al*. conducted a similar intercross between Tg26 and the same BAC-*APOL1* transgenic lines.^26^ This study was different from our study in that the BAC-*APOL1* mice were not on the FVB genetic background but were on 129SvJ. This produced offspring with a mixed genetic background that resulted in significant attenuation of the HIVAN phenotype. Interestingly, on this attenuating mixed background, G1 coexpression significantly exacerbated CKD. Thus, our observations appear consistent with this previous study: On the FVB/N background, G1 coexpression intensified the Tg26 failure-to-thrive phenotype in young animals, whereas on the mixed genetic background of the study by Yoshida *et al*.^26^, G1 intensified the CKD phenotype in adults. In addition, adult CKD outcomes from our G0/Tg26 intercross, which were not affected by the pregnancy complications, showed no improvement with G0 coexpression, and therefore, would not support the loss-of-function mechanism. The previous study by Yoshida *et al*.^26^ was unable to test the loss-of-function mechanism because the mixed genetic background did not result in any kidney disease in Tg26 animals on which improvement could be assessed in dual transgenic animals. In composite, the previous study design by Yoshida *et al*.^26^ was able to better test the exacerbating role of G1, and our study design here was able to better test the protective role of G0. Both studies support the gain-of-function mechanism for *APOL1* risk variants in driving CKD.

We previously reported that G0 appeared to have a protective effect on kidney disease in a similar transgenic intercrossing experiment.^30^ However, this study was technically different, using transgenic mouse models in which *APOL1* expression was controlled by the *Nphs1* promoter, restricting *APOL1* expression to podocytes. This methodologic difference could be informative as to the role of other kidney cell types that express *APOL1*. As shown here and as reported by others,^50^ both vascular endothelial cells and infiltrating immune cells express comparable levels of *APOL1*. It is possible *APOL1* expression in these other cell types may be underlying the deleterious effect of the risk variants. A second difference between these two studies is that our previous study used a congenic of the Tg26 mouse model which develops less severe kidney disease.^24^ It is possible that the combination of a non-native *APOL1* expression pattern with the milder kidney disease model contributed to these differences in outcomes. Of note, in both our previous study and the results presented here, the mice expressing only G0 had the highest podocyte density of all mice in the study. It is unclear why G0 expression would be associated with greater podocyte density, but it is possible this may have contributed to resilience to a milder disease stressor in our previous study.

In addition to the confounding issues from the pregnancy phenotype already discussed, other study limitations include shortcomings of the mouse models. The Tg26 HIVAN model does not replicate all aspects of the viral lifecycle, thus potential APOL1 interactions with initial events in infection could not be tested. The creation of the *APOL1* mouse models used a random insertion method that cannot control for transgene insertion site or copy number, thus differences in phenotypes between mouse lines due to host gene disruptions cannot be excluded. The *APOL1* mouse models also used engineered transgenes on the G0 EMR haplotype and may not fully reflect the function of native risk variant haplotypes.^51,52^ Owing to the pregnancy and neonatal complications, obtaining G1 dual transgenics was problematic, and this genotype was underpowered for sex differences in the adult phenotyping.

In summary, the absence of a mitigating effect by G0 in a mouse model of HIVAN potentially supports a gain-of-function mechanism. In addition, the pregnancy complications associated with G1 resulted in kidney developmental changes including reduced podocyte and glomerular densities, resulting in increased albumin excretion and worsened pathology. Because similar pregnancy complications in humans are known to be detrimental to kidney development and increase the future risk of CKD, these observations suggest a second mechanism by which *APOL1* variant alleles may contribute to the overall risk for CKD later in life.

## Supplementary Material

## Data Availability

Original data generated for the study are or will be made available in a public access repository upon publication. Data Type: Raw Data/Source Data. Repository Name: Zenodo. Linkable Citation: Source data have been deposited at Zenodo (doi: 10.5281/zenodo.19457719).

## Declarative Statements

All animal experiments were conducted in accordance with the NIH Guide for the Care and Use of Laboratory Animals or an equivalent standard that meets or exceeds the ethical and welfare requirements outlined in the NIH Guide. All protocols were approved by the appropriate institutional animal care and use committee.

## Supplemental Material

Supplemental Figure 1. BAC-*APOL1*-G1 mouse model on the FVB/*N* background.

Supplemental Figure 2. Genotype differences in pup, fetus and placental weights, and litter size.

Supplemental Figure 3. *APOL1* in human and mouse placenta is expressed in similar cell types and distribution.

Supplemental Figure 4. Tg26 HIV transgene expression in adult mouse kidney.

Supplemental Figure 5. *APOL1* and Tg26 HIV transgene expression in the developing mouse kidney.

Supplemental Figure 6. Kidney histopathology comparison for Tg26 and dual transgenic mice in the G0 and G1 intercross.

Supplemental Table 1. Antibodies and key reagents list.
