# Supplementary material for: Impact of Apolipoprotein L1-Associated Pregnancy Complications on Kidney Disease Phenotypes in a HIV-Associated Nephropathy Mouse Model
Source: Kidney360. 2026 Jul 2;7(9):1943–55. doi: 10.34067/KID.0000001253 (PMC13641498; doi:10.34067/KID.0000001253)
Supplement: Supplementary file 1 [file kidney360-7-1943-s001.pdf]

ASN American Society of Nephrology | JASN Journal of the American Society of Nephrology | KIDNEY360 Accessing Our World From Every Angle | CJASN Clinical Journal of the American Society of Nephrology

ASN Journal Disclosure Form

As per ASN journal policy, I have disclosed any financial relationships or commitments I have held in the past 36 months as included below. I have listed my Current Employer below to indicate there is a relationship requiring disclosure. If no relationship exists, my Current Employer is not listed.

T. Azhibekov reports the following:
Employer: Cleveland Clinic

I understand that the information above will be published within the journal article, if accepted, and that failure to comply and/or to accurately and completely report the potential financial conflicts of interest could lead to the following: 1) Prior to publication, article rejection, or 2) Post-publication, sanctions ranging from, but not limited to, issuing a correction, reporting the inaccurate information to the authors' institution, banning authors from submitting work to ASN journals for varying lengths of time, and/or retraction of the published work.

Name: Timur Azhibekov
Manuscript ID: K360-2026-000204
Manuscript Title: Impact of APOL1-associated pregnancy complications on kidney disease phenotypes in a HIVAN mouse model
Date of Completion: April 7, 2026
Disclosure Updated Date: March 29, 2026

ASN American Society of Nephrology · JASN Journal of the American Society of Nephrology · KIDNEY360 Accessing Our World From Every Angle · CJASN Clinical Journal of the American Society of Nephrology

ASN Journal Disclosure Form

As per ASN journal policy, I have disclosed any financial relationships or commitments I have held in the past 36 months as included below. I have listed my Current Employer below to indicate there is a relationship requiring disclosure. If no relationship exists, my Current Employer is not listed.

K. Bartolomeo reports the following:
Employer: Cleveland Clinic Foundation

I understand that the information above will be published within the journal article, if accepted, and that failure to comply and/or to accurately and completely report the potential financial conflicts of interest could lead to the following: 1) Prior to publication, article rejection, or 2) Post-publication, sanctions ranging from, but not limited to, issuing a correction, reporting the inaccurate information to the authors' institution, banning authors from submitting work to ASN journals for varying lengths of time, and/or retraction of the published work.

Name: Korey Bartolomeo
Manuscript ID: K360-2026-000204
Manuscript Title: Impact of APOL1-associated pregnancy complications on kidney disease phenotypes in a HIVAN mouse model
Date of Completion: April 2, 2026
Disclosure Updated Date: April 2, 2026

ASN American Society of Nephrology | JASN Journal of the American Society of Nephrology | KIDNEY360 Accessing Our World From Every Angle | CJASN Clinical Journal of the American Society of Nephrology

ASN Journal Disclosure Form

As per ASN journal policy, I have disclosed any financial relationships or commitments I have held in the past 36 months as included below. I have listed my Current Employer below to indicate there is a relationship requiring disclosure. If no relationship exists, my Current Employer is not listed.

L. Bruggeman reports the following:
Employer: Cleveland Clinic; and Patents or Royalties: Sanofi-Genzyme.

I understand that the information above will be published within the journal article, if accepted, and that failure to comply and/or to accurately and completely report the potential financial conflicts of interest could lead to the following: 1) Prior to publication, article rejection, or 2) Post-publication, sanctions ranging from, but not limited to, issuing a correction, reporting the inaccurate information to the authors' institution, banning authors from submitting work to ASN journals for varying lengths of time, and/or retraction of the published work.

Name: Leslie A. Bruggeman
Manuscript ID: K360-2026-000204
Manuscript Title: Impact of APOL1-associated pregnancy complications on kidney disease phenotypes in a HIVAN mouse model
Date of Completion: March 27, 2026
Disclosure Updated Date: March 27, 2026

ASN American Society of Nephrology | JASN Journal of the American Society of Nephrology | KIDNEY360 Accessing Our World From Every Angle | CJASN Clinical Journal of the American Society of Nephrology

ASN Journal Disclosure Form

As per ASN journal policy, I have disclosed any financial relationships or commitments I have held in the past 36 months as included below. I have listed my Current Employer below to indicate there is a relationship requiring disclosure. If no relationship exists, my Current Employer is not listed.

C. Huang reports the following:
Employer: Cleveland clinic foundation

I understand that the information above will be published within the journal article, if accepted, and that failure to comply and/or to accurately and completely report the potential financial conflicts of interest could lead to the following: 1) Prior to publication, article rejection, or 2) Post-publication, sanctions ranging from, but not limited to, issuing a correction, reporting the inaccurate information to the authors' institution, banning authors from submitting work to ASN journals for varying lengths of time, and/or retraction of the published work.

Name: Chunfa Huang
Manuscript ID: K360-2026-000204
Manuscript Title: Impact of APOL1-associated pregnancy complications on kidney disease phenotypes in a HIVAN mouse model
Date of Completion: April 7, 2026
Disclosure Updated Date: April 2, 2026

ASN American Society of Nephrology
JASN Journal of the American Society of Nephrology
KIDNEY360 Accessing Our World From Every Angle
CJASN Clinical Journal of the American Society of Nephrology

ASN Journal Disclosure Form

As per ASN journal policy, I have disclosed any financial relationships or commitments I have held in the past 36 months as included below. I have listed my Current Employer below to indicate there is a relationship requiring disclosure. If no relationship exists, my Current Employer is not listed.

J. Nguyen reports the following:
Employer: Cleveland Clinic; Consultancy: SamanTree; Advisory or Leadership Role: Johnson&Johnson; and Speakers Bureau: Johnson&Johnson.

I understand that the information above will be published within the journal article, if accepted, and that failure to comply and/or to accurately and completely report the potential financial conflicts of interest could lead to the following: 1) Prior to publication, article rejection, or 2) Post-publication, sanctions ranging from, but not limited to, issuing a correction, reporting the inaccurate information to the authors' institution, banning authors from submitting work to ASN journals for varying lengths of time, and/or retraction of the published work.

Name: Jane K. Nguyen
Manuscript ID: K360-2026-000204R2
Manuscript Title: Impact of APOL1-associated pregnancy complications on kidney disease phenotypes in a HIVAN mouse model
Date of Completion: May 11, 2026
Disclosure Updated Date: April 3, 2026

ASN
American Society of Nephrology
JASN
Journal of the American Society of Nephrology
KIDNEY360
Accessing Our World From Every Angle
CJASN
Clinical Journal of the American Society of Nephrology

ASN Journal Disclosure Form

As per ASN journal policy, I have disclosed any financial relationships or commitments I have held in the past 36 months as included below. I have listed my Current Employer below to indicate there is a relationship requiring disclosure. If no relationship exists, my Current Employer is not listed.

J. O'Toole reports the following:
Employer: Cleveland Clinic; Research Funding: Clinical trials sponsored by NIH.; And clinical trials by Vertex, Maze, Novartis, Chinnock, Boehringer Ingelheim no salary support.; Patents or Royalties: Sanofi-Genzyme ~$3300 received for licensing fee for use of transgenic mouse model; and Other Interests or Relationships: NIH grant support.

I understand that the information above will be published within the journal article, if accepted, and that failure to comply and/or to accurately and completely report the potential financial conflicts of interest could lead to the following: 1) Prior to publication, article rejection, or 2) Post-publication, sanctions ranging from, but not limited to, issuing a correction, reporting the inaccurate information to the authors' institution, banning authors from submitting work to ASN journals for varying lengths of time, and/or retraction of the published work.

Name: John F. O'Toole
Manuscript ID: K360-2026-000204
Manuscript Title: Impact of APOL1-associated pregnancy complications on kidney disease phenotypes in a HIVAN mouse model
Date of Completion: April 3, 2026
Disclosure Updated Date: April 3, 2026

ASN Journal Disclosure Form

As per ASN journal policy, I have disclosed any financial relationships or commitments I have held in the past 36 months as included below. I have listed my Current Employer below to indicate there is a relationship requiring disclosure. If no relationship exists, my Current Employer is not listed.

J. Sedor reports the following:
Consultancy: MAZE (Relationship has not ended); Research Funding: MAZE (Relationship has not ended); Novartis (Relationship has not ended); Vertex (Relationship has not ended); and Patents or Royalties: Sanofi (Relationship has not ended).

I understand that the information above will be published within the journal article, if accepted, and that failure to comply and/or to accurately and completely report the potential financial conflicts of interest could lead to the following: 1) Prior to publication, article rejection, or 2) Post-publication, sanctions ranging from, but not limited to, issuing a correction, reporting the inaccurate information to the authors' institution, banning authors from submitting work to ASN journals for varying lengths of time, and/or retraction of the published work.

Name: John R. Sedor
Manuscript ID: K360-2026-000204
Manuscript Title: APOL1-associated pregnancy complications reduced podocyte density and exacerbated kidney disease phenotypes in a HIVAN mouse model
Date of Completion: March 30, 2026
Disclosure Updated Date: March 30, 2026

ASN American Society of Nephrology | JASN Journal of the American Society of Nephrology | KIDNEY360 Accessing Our World From Every Angle | CJASN Clinical Journal of the American Society of Nephrology

ASN Journal Disclosure Form

As per ASN journal policy, I have disclosed any financial relationships or commitments I have held in the past 36 months as included below. I have listed my Current Employer below to indicate there is a relationship requiring disclosure. If no relationship exists, my Current Employer is not listed.

O. Wessely reports the following:
Employer: Cleveland Clinic Foundation/Lerner Research Institute

I understand that the information above will be published within the journal article, if accepted, and that failure to comply and/or to accurately and completely report the potential financial conflicts of interest could lead to the following: 1) Prior to publication, article rejection, or 2) Post-publication, sanctions ranging from, but not limited to, issuing a correction, reporting the inaccurate information to the authors' institution, banning authors from submitting work to ASN journals for varying lengths of time, and/or retraction of the published work.

Name: Oliver Wessely
Manuscript ID: K360-2026-000204R2
Manuscript Title: Impact of APOL1-associated pregnancy complications on kidney disease phenotypes in a HIVAN mouse model
Date of Completion: April 26, 2026
Disclosure Updated Date: May 9, 2025

ASN American Society of Nephrology | JASN Journal of the American Society of Nephrology | KIDNEY360 Accessing Our World From Every Angle | CJASN Clinical Journal of the American Society of Nephrology

ASN Journal Disclosure Form

As per ASN journal policy, I have disclosed any financial relationships or commitments I have held in the past 36 months as included below. I have listed my Current Employer below to indicate there is a relationship requiring disclosure. If no relationship exists, my Current Employer is not listed.

Z. Wu reports the following:
Employer: Cleveland Clinic

I understand that the information above will be published within the journal article, if accepted, and that failure to comply and/or to accurately and completely report the potential financial conflicts of interest could lead to the following: 1) Prior to publication, article rejection, or 2) Post-publication, sanctions ranging from, but not limited to, issuing a correction, reporting the inaccurate information to the authors' institution, banning authors from submitting work to ASN journals for varying lengths of time, and/or retraction of the published work.

Name: Zhenzhen Wu
Manuscript ID: K360-2026-000204
Manuscript Title: Impact of APOL1-associated pregnancy complications on kidney disease phenotypes in a HIVAN mouse model
Date of Completion: April 7, 2026
Disclosure Updated Date: March 30, 2026
