# Supplementary material for: Impact of Apolipoprotein L1-Associated Pregnancy Complications on Kidney Disease Phenotypes in a HIV-Associated Nephropathy Mouse Model
Source: Kidney360. 2026 Jul 2;7(9):1943–55. doi: 10.34067/KID.0000001253 (PMC13641498; doi:10.34067/KID.0000001253)
Supplement: Supplementary file 2 [file kidney360-7-1943-s002.pdf]

Supplementary Material

Supplemental Methods

Mouse models
Transgene insertion site
Fibrosis quantification by sirius red staining
mRNA *in situ* hybridization
Supplemental references

Supplemental Figures

Figure S1. BAC-*APOL1*-G1 mouse model on the FVB/N background.

Figure S2. Genotype differences in pup, fetus and placental weights and litter size.

Figure S3. *APOL1* in human and mouse placenta is expressed in similar cell types and distribution.

Figure S4. Tg26 HIV transgene expression in adult mouse kidney.

Figure S5. *APOL1* and Tg26 HIV transgene expression in the developing mouse kidney.

Figure S6. Kidney histopathology comparison for Tg26 and dual transgenic mice in the G0 and G1 intercross.

Supplemental Table

Table S1. Antibodies and key reagents list

Supplemental Methods

Mouse models

All animal studies were reviewed and approved by the Cleveland Clinic Institutional Animal Care and Use Committee (IACUC) , under study number 2435. The Tg26 model of HIVAN was obtained from Jackson Laboratory in 2021 and is maintained on the FVB/NJ background. Transgenic mice expressing human bacterial artificial chromosomes (BACs) for *APOL1*-G0, *APOL1*-G1, or *APOL*-G2 were developed at Merck Research Labs and are distributed by Taconic (**Methods Table 1**). In the BAC transgene constructions, the G1 and G2 mutations were not from naturally occurring G1 or G2 alleles (EIK haplotype), but were engineered onto the G0 reference allele (EMR haplotype). All *APOL1* BAC transgenic lines were obtained on a 129/B6 background but for this study were backcrossed >10 generations to FVB/Nj prior to conducting the intercross with Tg26 to preserve the Tg26 phenotype which is dependent on the FVB/N background.

Methods Table 1. Mouse models used in this study.

Model/Strain	Source	Strain number	Strain designation
APOL1-G0	Taconic (Merck)	13017	129S1.Cg-Tg(APOL1)33_M04Mrl
APOL1-G1	Taconic (Merck)	13175	129S1;B6/J-Tg(APOL1*S342G,*I384M)F03Mrl
APOL1-G2	Taconic (Merck)	13022	129;B6-Tg(APOL1*NYK388K)85_M02Mrl
Tg26	Jackson Labs	022354	FVB/N-Tg(HIV)26Aln/PkltJ
FVB	Jackson Labs	001800	FVB/NJ

All animals were house in a specific pathogen-free facility in standard caging on ventilated racks. Animals were handled in a biological safety cabinet under aseptic conditions, provided with sterilized bedding, standard chow and water, and were given standard enrichment including group housing. Pain and distress were managed by preemptive and interventional euthanasia. Veterinary input was sought throughout the study regarding use of euthanasia. Study humane endpoints that required interventional euthanasia include end stage kidney disease (4+ proteinuria or physical evidence of peripheral edema) and signs of preeclampsia including seizures (hypersalivation, hyperactivity or repetitive motion, malaise). Preemptive euthanasia by 100 days of age was also mandated by the IACUC for general use of the BAC-*APOL1*-G1 line to eliminate "death as an endpoint" due to the shortened lifespan (described below) and also for all G1 breeder females after litter weaning to eliminate any potential pain and distress from long term effects of the preeclamptic pregnancy. Interventional euthanasia was also mandated by the IACUC for general use of the Tg26 line for the kidney disease phenotype (as described above) or for a skin lesion phenotype that was unrelated to this study. The Tg26 mouse model is known to develop hyperkeratotic skin lesions[1] that are generally benign but can become ulcerated. Interventional euthanasia was used if the skin lesions became ulcerated. Animals were monitored daily for these endpoints by research staff or veterinary technicians for body condition score and endpoints described above. Animals that were identified for euthanasia for reasons other than a study-defined kidney disease endpoint were excluded from the study. Tg26 mice were intercrossed with both *APOL1*-G0 and *APOL1*-G1 BAC lines using standard breeding practices in which Tg26 males were mated with *APOL1* females in all G0 intercrosses and all but two G1 intercrosses. This breeding strategy replicated the human inheritance since *APOL1* was expressed in both maternal and fetal tissues. When feasible, individual Tg26 males were used to

sire litters with both *APOL1*-G0 and G1 females, however, dams were not used for multiple pregnancies.

Blood pressures during pregnancy were recorded using tail cuff plethysmography (IITC, San Diego, CA). Female mice were acclimated to the recording system and then blood pressures were recorded every other day for three assessment periods: 1) one week prior to exposure to a male for mating (pre-pregnancy), 2) during pregnancy (starting 2 days after the appearance of a vaginal plug), and 3) one week post-partum.

In the course of backcrossing to FVB/N, the lifespan of the BAC-*APOL1*-G1 mice declined for unknown reasons (**Supplemental Figure S1A**). Attempts to identify a cause were unsuccessful largely due to the sudden onset of death, in which mice typically would not exhibit any prodrome or symptoms of impending death. Moribund/deceased mice (*n*=8) did not show evidence of kidney pathology, indicating death was not due to kidney disease (**Supplemental Figure S1B**). Analysis of healthy G1 mice did not reveal any obvious abnormalities. A prior report using these same BAC transgenic lines, but on the C57BL/6 background, reported 20% of the G1 animals intercrossed with an ApoE null mouse model exhibited congenital hydrocephalus, presenting with an enlarged braincase.[2] We did not observe congenital hydrocephalus in any of our G1 mice on the FVB/N background or in the Tg26 intercrosses. In addition, necropsy of brains from adult G1 mice were examined for enlargement of lateral ventricles as an indication of hydrocephalus onset after fusion of cranial sutures. There was no evidence of enlarged ventricles in adult G1 mice, even in moribund animals (**Supplemental Figure S1C-D**). According to Jackson Laboratories data,[3] a small percentage of C57BL/6 mice spontaneously develop congenital hydrocephalus for unknown reasons. It is possible this prior report of hydrocephalus in G1 mice may be enhanced due to intrinsic properties of the C57BL/6 background. In addition, this prior study also reported *APOL1* was expressed in choroid plexus epithelia which resulted in upregulation of solute transporters leading to overproduction of cerebrospinal fluid. In G1 mice on the FVB/N background, we did not observe any *APOL1* expression in the brain other than vascular endothelia (**Supplemental Figure S1C-G**).

Identification of BAC-APOL1-G0 transgene insertion site.

Genomic DNA (gDNA) was extracted from a freshly isolated spleen from a G0 mouse. The quality and concentration of the gDNA were determined by optical density 260nm/280nm ratio on a BioTek Synergy H1 microplate reader and by electrophoretic analysis on an agarose gel. The high-quality, high molecular weight gDNA was used for analyzing the breaking point region of the BAC *APOL1* transgene insertion using the GenomeWalker kit (Takara Bio USA) according to the manufacturer's instruction. The gDNA was digested individually with four different blunt-end restriction enzymes *Dra*I, *Eco*RV, *Pvu*II, and *Stu*I, followed by ligation to kit provided adapters. Two phase PCR was performed according to kit instructions using the adapter primers (AP1 and AP2) and nested gene specific primers (A2R9 and A2R10, **Methods Table 2**) designed to be near the predicted BAC end which included the 5'-end of the *APOL2* gene. PCR products were separated on 1.5% agarose gels, DNA bands were excised from the agarose gel, purified from the agarose using a Qiagen gel extraction kit, and PCR products were sequenced by Sanger sequencing using one of the PCR primers.

Methods Table 2. Primers used in G0 transgene insertion site

primer	sequence	Source
AP1	5'-GTAATACGACTCACTATAGGGC-3'	Takara Bio
AP2	5'-GTAATACGACTCACTATAGGGC-3'	Takara Bio
A2R9	5'-GTCTCTGAGGACAGGTGACTTGCAC-3'	custom
A2R10	5'-GGATGGGAGTGGGAGGAGTAGATGG-3'	custom
G0 confirm F1	5'-TGGAACTTGCTGCTTTATTGAC-3'	custom
G0 confirm F2	5'-GCACTTTGGGGAACACCTGAAC-3'	custom

Sequences were aligned to the mouse genome using the UCSC Genome Browser (genome.ucsc.edu) with build GRCm38/mm10. The insertion point of the human BAC G0 transgene was on mouse Chr2:146,313,130 (**Methods Figure 1A**). The insertion site was confirmed by generating new PCR primers near the insertion site in the mouse genome (**Methods Table 2**). Standard PCR (30 cycles with anneal temperature of 68°C using the Advantage 2 PCR kit, Takara Bio USA) with *Dra*I digested DNA using the following primer pairs: G0 confirm F1+ A2R10, G0 confirm F1+ A2R9, G0 confirm F2+ A2R10 or G0 confirm F2+ A2R9) produced the predicted fragment sizes of 248, 338, 326 and 380 bp respectively (**Methods Figure 1B**).

Methods Figure 1A. Insertion site sequence

```
GGCCCGGCGGTAATCTTCACTATGTTTTTTAACTGTATCCATGTGCCTGTGCCAG
CTAGTTAATGACCAGCTGCTCCCTTCATGTTCACTGGGACACTGAAGTTCACAGT
GCACTTTGGGGAACACCTGAACTGCTTCAGTGTCATAGCTGATGTCTGGAACTTG
CTGCTTTATTGACATAAGTCTGATATTTGAATTTTGAAAAACACTAAATACTGTA
ACCAGTCCTAATTTTGAGTACTAATTGCATTTATTCATTTACCAAACAAACATTT
CCTGTGGACTGTCTGCCAAGTTCAGTGCTAGACGCTGGGGCTACTGCATCACCTC
AGGGACCCAGGAGCCCAGTCCTTGTTCACACATTTCAGAGATAATACCCACTGAC
GCCCTGCTTCGGTGCCTCCTGGAGCCCATCTACTCCTCCCACCTCCCATCCACC
```

adapter sequence
mouse genomic sequence
human *APOL2* (transgene) sequence

Methods Figure 1B.

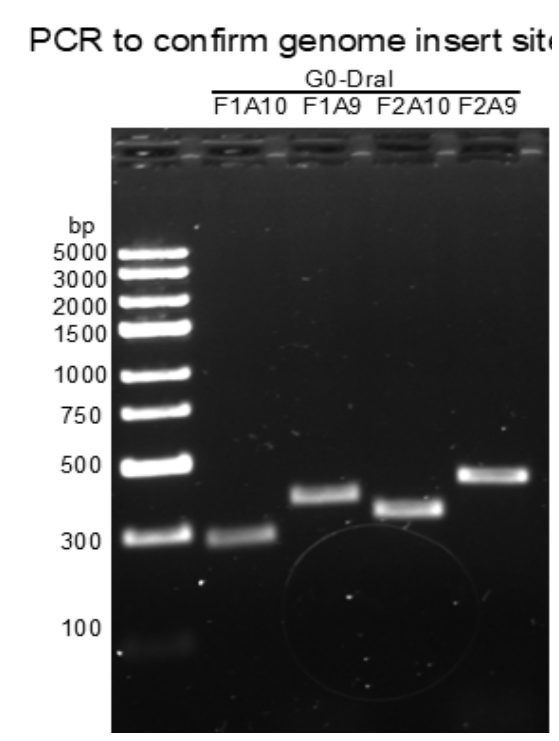

Based on this insertion site analysis, the original description of the BAC construct used to create the *APOL1* G0, G1 and G2 transgenic mice, which was reported to contain exons 1-3 of the adjacent *APOL2* gene, was not accurate for the G0 transgene. The G0 BAC transgene only includes exon 1 of the *APOL2* gene. In addition, this PCR-based analysis also identified there is more than one copy of the BAC transgene at this insertion site, concatenated in a head-to-tail orientation. How many copies of the transgene present in the G0 line was not determined, but there are, at minimum, two copies in this single insertion site. The insertion site is located in intron 38 of the *Ralgapa2* gene. We did not examine expression of the *Ralgapa2* gene, however, a recent report using these same BAC G0 and G1 transgenic lines performed several genome wide expression studies.[4] In this study, the expression level of the *Ralgapa2* gene was significantly increased in both podocytes and endothelial cells in the G0 mice compared to G1 mice. It is important to reiterate that the G0 transgenic line has no phenotype and is hemizygous for the transgene (transgene is carried on only one chromosome) in which the other allele of the *Ralgapa2* gene is not affected by the transgene insertion.

The transgene insertion site of the BAC-*APOL1*-G1 mice has been previously reported in a noncoding region of Chromosome 13 and was reported to not affect the expression level of neighboring genes.[2]

Fibrosis quantification by sirius red staining

Full sagittal sections (4μm thickness) of each kidney were deparaffinized and stained for 1 hour with picro-sirius red solution (Abcam) followed by two washes in acidified water for 1 minute each. Slides were dehydrated with dips in 100% ethanol and then xylene before mounting in Permount (Fisher). Light micrograph images (TIFF format, 1024 x 1280 pixels, 72 pixels/inch) at 10X magnification were collected on a Nikon Eclipse 55i microscope using Nikon Digital Sight 10 frame capture and NIS Elements software (Nikon). Raw images were imported into ImageJ at 8bit resolution. Image size and pixel density were entered to set scale. Image was converted to greyscale, and the threshold was empirically set optimizing for clearest resolution of structures and then standardized for all images. Measurements of regions stained by sirius red were captured as percent area stained. An average of 5 images were captured from cortical regions for each kidney for estimates of fibrotic tissue area.

mRNA in situ hybridization

The ACDBio (Bio-Techne) RNAscope manual chromogenic method was used for either single probes (RNAscope 2.5HD Manual kit, Fast Red) or for two probes (RNAscope 2.5HD Duplex Assay, Fast Red and Fast Green) following the manufacturer's instructions. All tissues were fixed in neutral buffered formalin overnight at 4°C and embedded in paraffin using standard procedures. We used low melting point paraffin (Surgipath Blue Ribbon, Leica Biosystems). Paraffin blocks that were used for RNA analysis were store at -20°C. Cut sections (4μm) were mounted on Fisher Superfrost Plus 1x3 inch glass slides and dried flat overnight on a slide warmer (42°C). Slides should be used immediately, but if necessary, can be stored at -20°C until use, but storage of cut slides >1 month results in significant loss in RNA detection.

Day 1: Immediately before starting the *in situ* procedure, slides were laid flat and baked for 1 hour at 60°C. Slides were deparaffinized using standard methods (submerge slides in fresh Xylenes twice followed by twice in fresh 100% ethanol, 5 min each) and air dried. Slides were pretreated per kit instructions for the manual target retrieval method as follows: Dried slides were laid flat and kit provided hydrogen peroxide was applied to cover the section and incubated for 10 min at room temperature followed by two, 1 min washes in distilled water. Slides were submerged in 98-102°C kit provided Target Retrieval Reagent and incubated under a mild boil for 15 min followed by two, 1 min washes in distilled water, a 1 min wash in 100% ethanol and air dried. On the dried slides, tissue sections were circled with a hydrophobic barrier pen (ImmEdge Pen, Vector Labs) and slides were stored overnight at room temperature in an air tight container.

Day 2: Dried slides were laid flat in a humidified chamber and kit provided Protease Plus Reagent was added to cover sections and incubated at 40°C for 30 min. Slides were washed twice for 1 min each in distilled water. Without letting the slides dry, slides were laid flat in a humidified chamber, and pre-warmed probes were added to cover sections and incubated for 2 h at 40°C.

For singlepex assay, the ready-to-use channel 1 probes were applied to sections, for the duplex assay, channel 1 and channel 2 probes were mixed together as instructed and similarly applied to sections. After hybridization, slides were submerged in kit provided 1X wash buffer and washed twice for 2 min each with shaking. The kit provided instructions were followed as written to process slides through the six step (singleplex) or ten step (duplex) amplification process using kit provided reagents. Kit instructions were also followed as written to prepare the chromogenic detection reagents. After amplification steps, chromogenic detection reagents were applied to sections and incubated protected from light for 10 min at room temperature. For singleplex, this is Fast Red and for duplex this is Fast Green for channel 1 and Fast Red for channel 2 (note Fast Red is used for different channel probes in the two assays). Slides were washed twice in water and counterstained with 5 dips in hematoxylin (Gill #3, Sigma) followed by 10 dips in tap water, 10 dips in 0.02% ammonia water, and 10 dips in fresh tap water. Slides were dried in a 60°C oven for 15 min. Sections were mounted under coverslips using EcoMount (Bio-Techne).

Light micrograph images were collected on a Nikon Eclipse 55i microscope using Nikon Digital Sight 10 frame capture and NIS Elements software (Nikon). Images were analyzed for quantification using the same process as described for sirius red staining using Image J.

Supplemental references.

1. Kopp JB, Rooney JF, Wohlenberg C, Dorfman N, Marinos NJ, Bryant JL, Katz SI, Notkins AL, Klotman PE: Cutaneous disorders and viral gene expression in HIV-1 transgenic mice. *AIDS Res Human Retroviruses* Mar 1993;9(3):267-275. doi:10.1089/aid.1993.9.267

2. Yoshida T, Yang ZH, Ashida S, Yu ZX, Shrivastav S, Rojulpote KV, Bahar P, Nguyen D, Springer DA, Munasinghe J, Starost MF, Hoffmann VJ, Rosenberg AZ, Bielekova B, Wen H, Remaley AT, Kopp JB: Apolipoprotein-L1 G1 variant contributes to hydrocephalus but not to atherosclerosis in apolipoprotein-E knock-out mice. *Clin Sci (Lond)* May 27 2025;doi:10.1042/cs20255324

3. Jackson Laboratories: Hydrocephalus in laboratory mice. *JAX Notes* 2003;49: https://www.jax.org/jaxnotes/archive/490f.

4. Yoshida T, Latt KZ, Santo BA, Shrivastav S, Zhao Y, Fenaroli P, Chung JY, Hewitt SM, Tutino VM, Sarder P, Rosenberg AZ, Winkler CA, Kopp JB: Single-Cell Transcriptional Signatures of Glomerular Disease in Transgenic Mice with APOL1 Variants. *J Am Soc Nephrol* Aug 1 2024;35(8):1058-1075. doi:10.1681/asn.0000000000000370

5. Blessing NA, Wu Z, Madhavan SM, Choy JW, Chen M, Shin MK, Hoek M, Sedor JR, O'Toole JF, Bruggeman LA: Lack of APOL1 in proximal tubules of normal human kidneys and proteinuric APOL1 transgenic mouse kidneys. *PloS One* 2021;16(6):e0253197. doi:10.1371/journal.pone.0253197

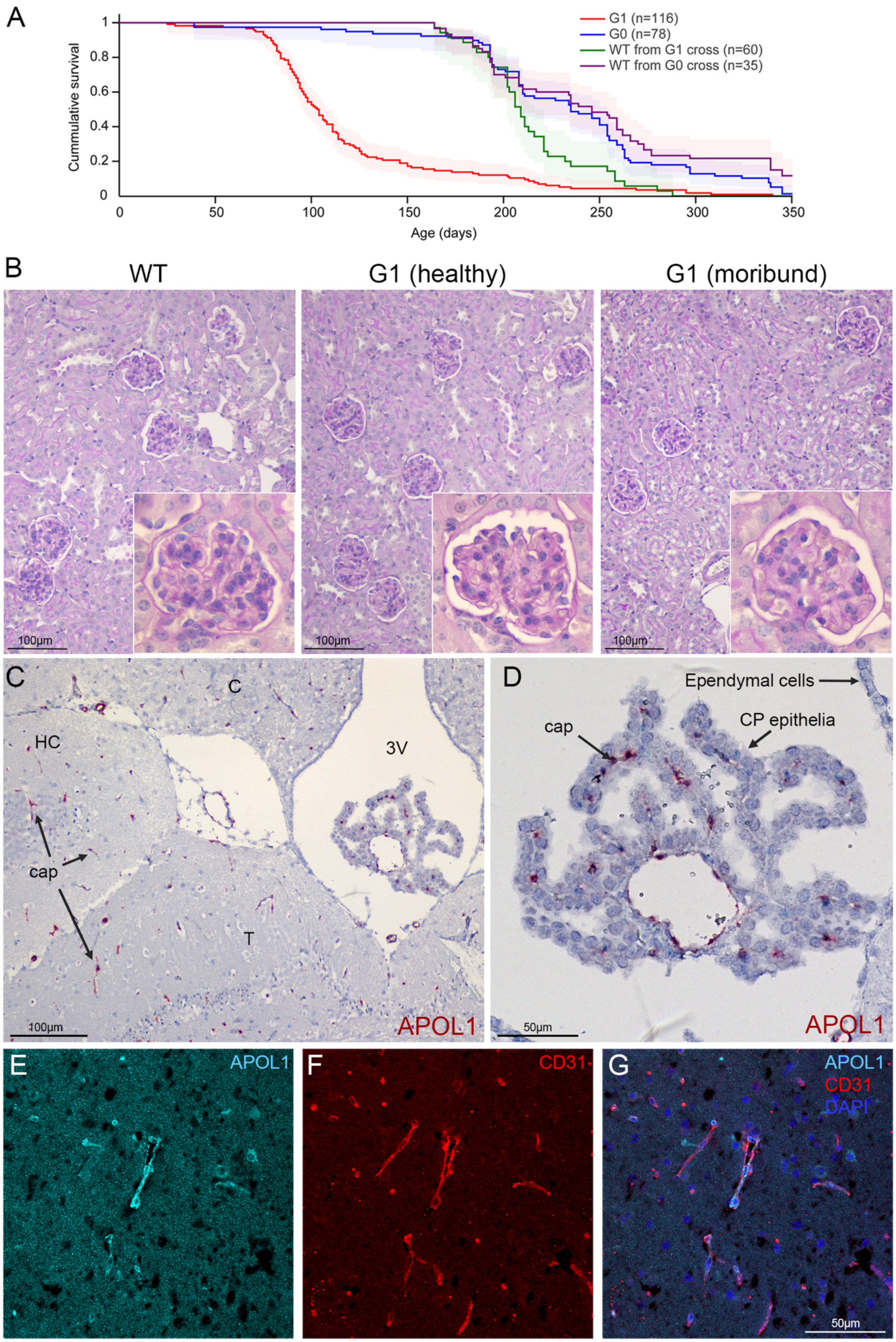

Supplemental Figure S1.

Supplemental Figure S1. **BAC-*APOL1*-G1 mouse model on the FVB/N background. A**. Kaplan Meier curve of survival of BAC-*APOL1*-G1 mice on the FVB/N background was significantly different than BAC-*APOL1*-G0 (log rank test $p=10^{-16}$). The survival curves for the G0 and WT mice from both crosses includes healthy animals that were euthanized as controls or as retired breeders. **B**. Comparison of kidney histopathology (PAS stain) from littermate females including a wildtype (WT) mouse and two G1 transgenics, one of which was moribund. All were euthanized at the same age (114 days). There were no histopathological findings that would indicate kidney disease was the cause of the G1 early deaths. **C-G**. Coronal section of G1 mouse brain examined for *APOL1* expression using RNA *in situ* hybridization and immunofluorescence. **C-D**. *APOL1* expression in brain was only observed in endothelial cells of capillary (cap) and larger vessel with no expression in choroid plexus (CP) or ependymal epithelia, the cell types that produce cerebrospinal fluid. **E-G.** Expression of APOL1 protein by immunofluorescence confirmed expression was in CD31 positive endothelial cells (a region of the cortex is shown, DAPI as nuclear stain). The APOL1 antibody used here has some technical limitations including moderate sensitive to detect APOL1 and some off target labeling of glomerular parietal cells, but it did accurately label endothelial cells in the kidney (quality control of this and other commercial APOL1 antibodies was done in our prior publication[5]). cap, capillary; C, cerebral cortex; CP, choroid plexus; HC, hippocampus; T, thalamus; 3V, third ventricle.

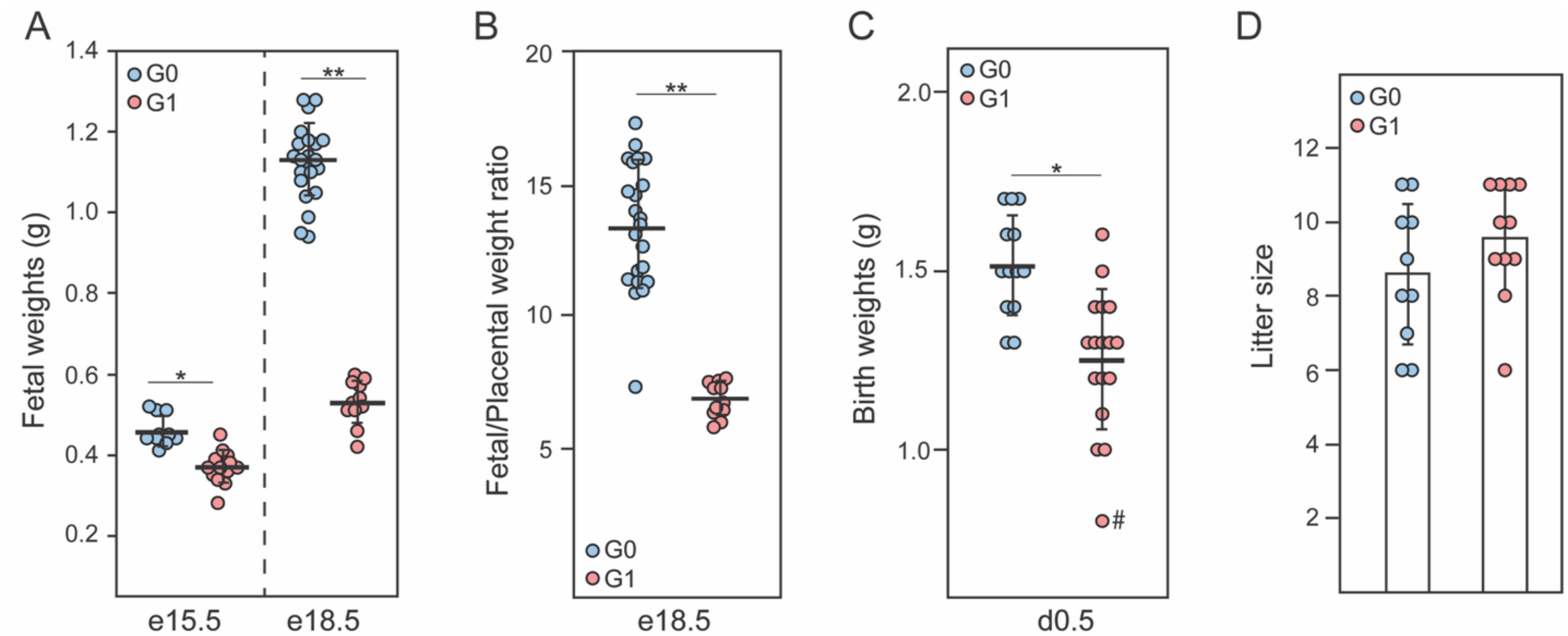

Supplemental Figure S2. Genotype differences in pup, fetus and placental weights and litter size. A. Fetal weights at embryonic day (e) 15.5 and e18.5 were significantly different, with G1 litters lower than G0 (*n*=3 litters each genotype). **B**. At near full term pregnancy (e18.5), fetal/placental weight ratios were significantly different between G0 and G1 litters. **C**. Newborn live and stillborn (#) pup weights on day (d) 0.5 were also lower in G1. D. Average litter size (#pups/litter) was not different (G0 *n*=10 litters, G1 *n*=11 litters). Data are mean±standard deviation, comparisons by t test *$p<0.05$, **$p<0.01$.

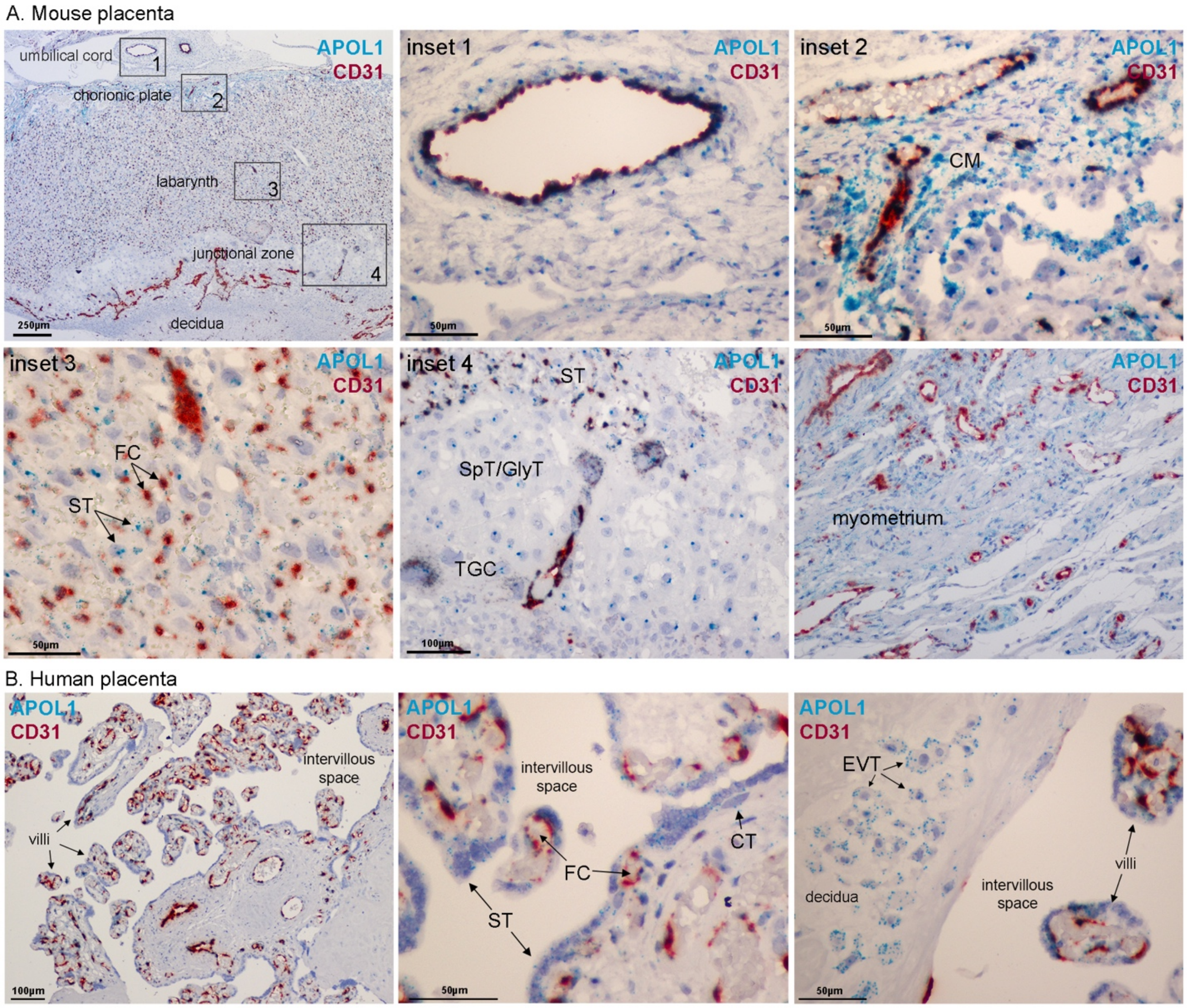

Supplemental Figure S3. ***APOL1* in human and mouse placenta is expressed in similar cell types and distribution.** *APOL1* and *CD31* expression was examined using a two probe *in situ* hybridization method and counterstain with hematoxylin. **A**. Structure of mouse placenta includes the fetal-derived labyrinth and junctional zone and the maternal-derived decidua and myometrium. *APOL1* expression was abundant in both maternal and fetal cells. Higher magnification insets are numbered. **B**. Villous structure of a term human placenta. CM, chorionic mesenchyme; CT, cytotrophoblasts; EVT, extravillous trophoblasts; FC, fetal capillary; GlyT, glycogen trophoblasts; SpT, spongiotrophoblasts; ST, syncytiotrophoblasts; TGC, trophoblast giant cells.

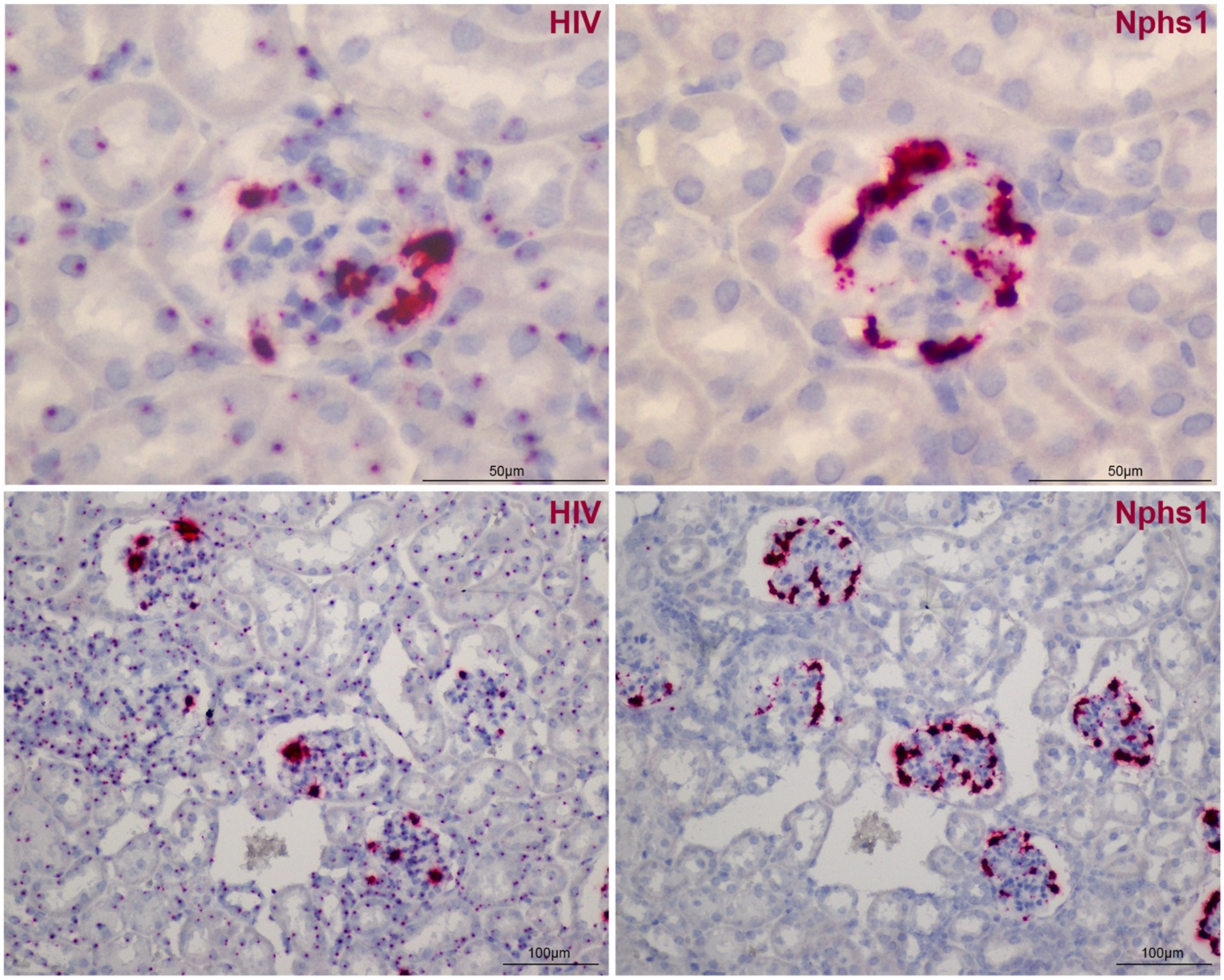

Supplemental Figure S4. Tg26 HIV transgene expression in adult mouse kidney. Expression of the Tg26 HIV transgene and the podocyte marker *Nphs1* (Nephrin) by RNA *in situ* hybridization. **A**. Serial sections of a mouse with early stage kidney disease at 40X (top) and 20X (bottom) magnification for HIV and *Nphs1* expression. HIV gene expression was detected in all epithelial cell types including tubular epithelia, parietal cells and podocytes.

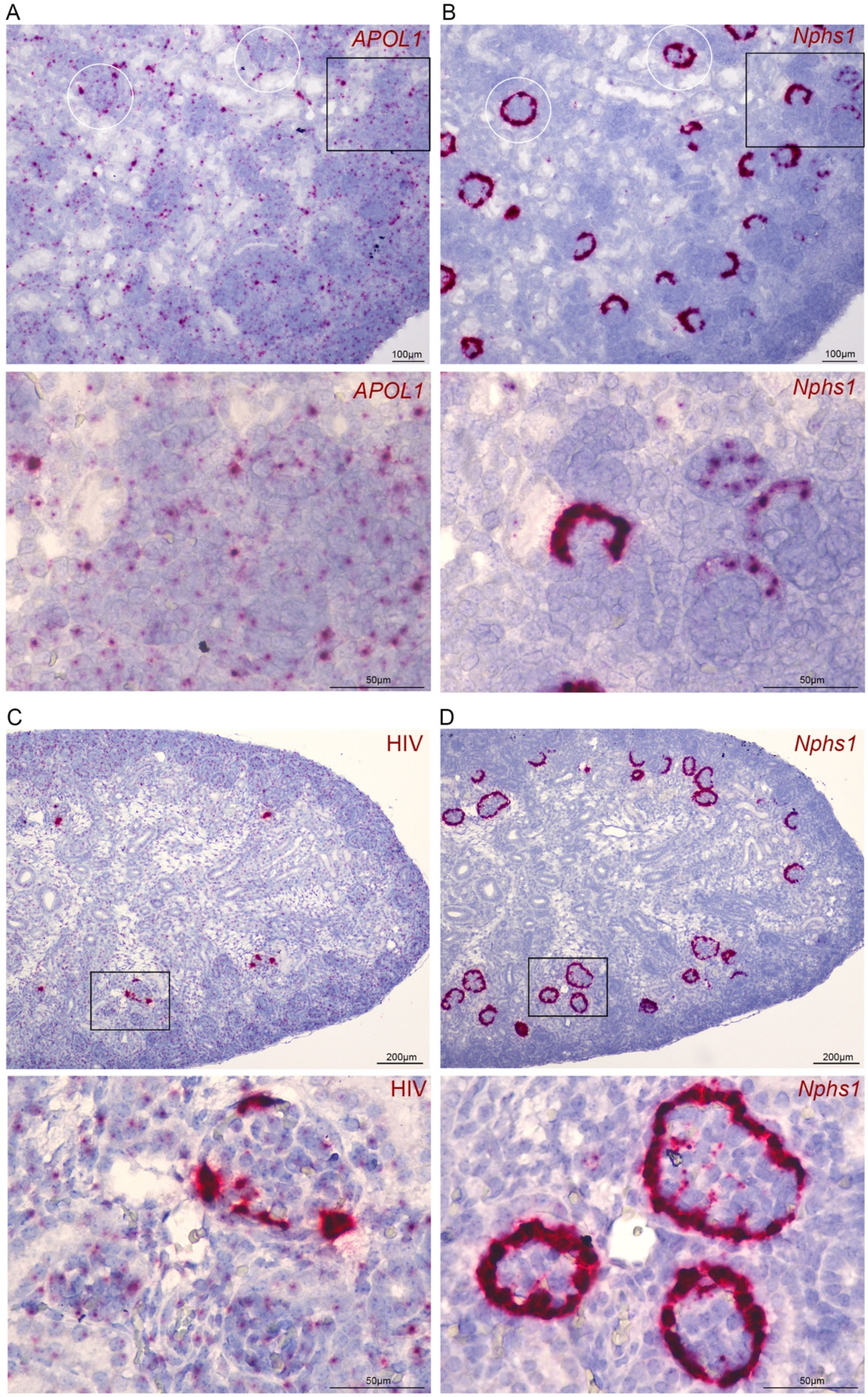

Supplemental Figure S5. *APOL1* and Tg26 HIV transgene expression in the developing mouse kidney. Cell types expressing *APOL1* and HIV were determined by mRNA *in situ*

hybridization in fetal mouse kidneys (e18.5). **A-B**. *APOL1* expression was only evident at low levels in sporadic cells and was absent from developing podocytes which were identified in a serial section with *Nphs1* expression (panel B). Circles denote the same glomerulus in serial sections. Inset boxes shown in higher magnification below image. **C-D.** HIV transgene expression was strong in podocytes in deeper, more mature glomeruli, also identified by co-localization with *Nphs1* in a serial section (panel D). Inset boxes shown in higher magnification below image.

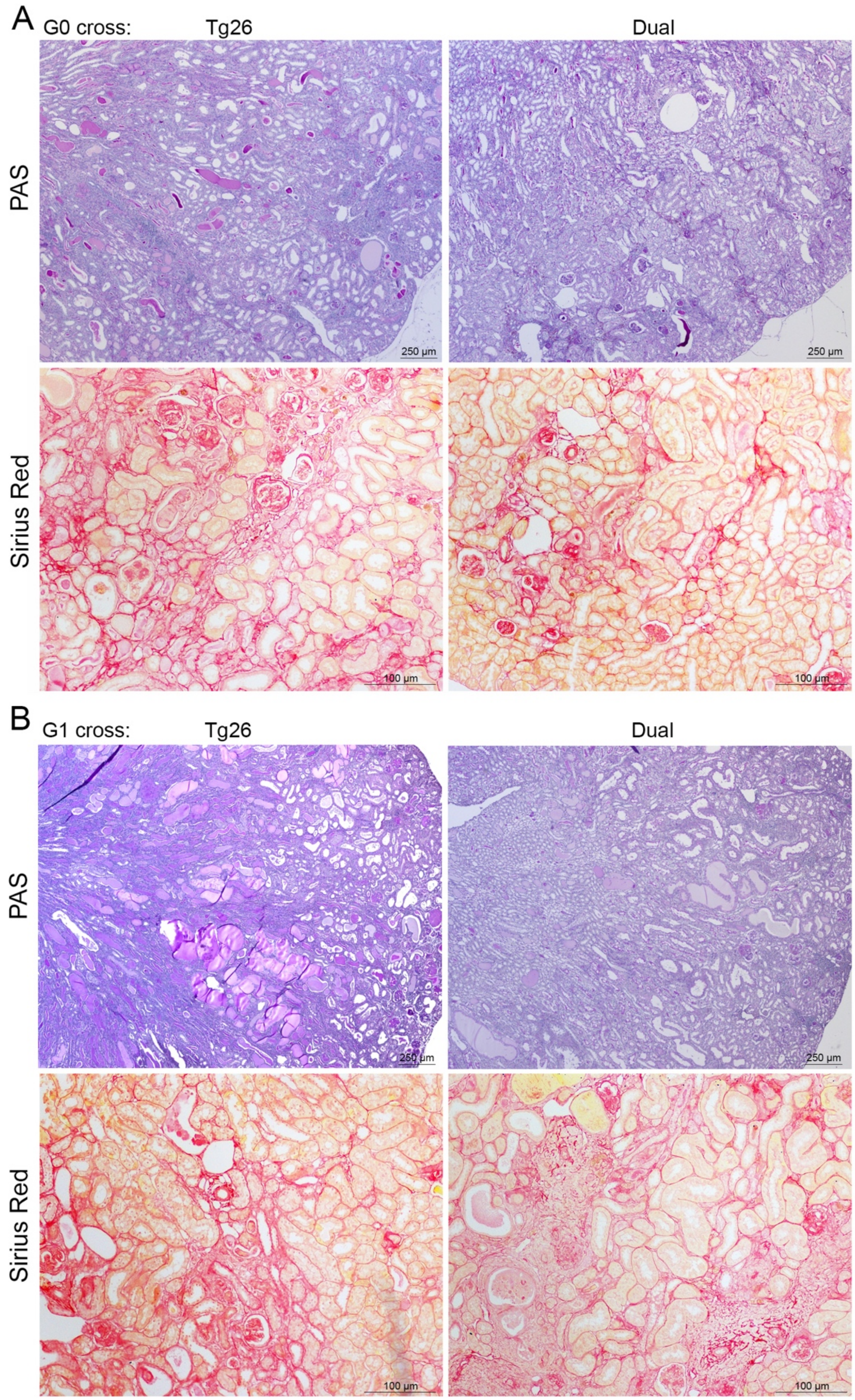

Supplemental Figure S6. Examples of kidney histopathology comparison for Tg26 and dual transgenic mice in the G0 and G1 intercross. PAS stain was used for scoring glomerular and tubulointerstitial lesions and Sirius red staining to score fibrosis. Quantification of these data is presented in Table 1. **A**. Comparison of the Tg26 and dual transgenic from the G0 intercross. **B**. Comparison of the Tg26 and dual transgenic mice from the G1 intercross.

Supplemental Table S1. Antibodies and key reagents list

Antibodies	Company & Catalog #	Assay	Dilution
Synaptopodin	Progen clone G1D4 365194	immunohistochemistry	1:10
WT-1	Proteintech 12609-1-AP	immunohistochemistry	1:400
Horse anti-rabbit AP	Vector labs MP-5401	immunohistochemistry	undiluted
Horse anti-mouse HRP	Vector Labs MP-7402	immunohistochemistry	undiluted
APOL1	Sigma (HPA018885) lot E105260*	Immunofluorescence	1:200
CD31/PECAM	BD pharmingen 550274 (mouse)	Immunofluorescence	1:200
CD31/PECAM	R&D AF3628 (human)	Immunofluorescence	1:200
anti-rabbit Cy3	Jackson Immuno Research 111-165-144	Immunofluorescence	1:400
anti-mouse Alex488	Invitrogen A11029	Immunofluorescence	1:400
anti-goat Cy5	Jackson Immuno Research 115-175-150	Immunofluorescence	1:400
anti-rat Cy5	Jackson Immuno Research 112-175-143	Immunofluorescence	1:400
**Chemicals**			
Sirius Red	Abcam AB246832		
Permount	Fisher SP15-100		
EcoMount	ACDbio 320409		
**In situ hybridization**			
RNAscope 2.5HD Manual kit (red)	ACDbio 322360		
RNAscope 2.5HD Duplex Assay	ACDbio 322430		
Wash buffer and reagents	ACDbio 310091		
Target retrieval reagents	ACDbio 322000		
H_2O_2 and Protease Plus	ACDbio 322330		
human APOL1	ACDbio Hs-APOL1-No-XMm 459791		
murine Nphs1	ACDbio Mm-Nphs1 433571		
murine CD31	ACDbio Mm-Pecam1 316721-C2		
HIV	ACDbio HIV-nongagpol 317711		

*lot no longer for sale by manufacturer
